# Signatures of the Evolution of Parthenogenesis and Cryptobiosis in the Genomes of Panagrolaimid Nematodes

**DOI:** 10.1016/j.isci.2019.10.039

**Published:** 2019-10-24

**Authors:** Philipp H. Schiffer, Etienne G.J. Danchin, Ann M. Burnell, Christopher J. Creevey, Simon Wong, Ilona Dix, Georgina O'Mahony, Bridget A. Culleton, Corinne Rancurel, Gary Stier, Elizabeth A. Martínez-Salazar, Aleksandra Marconi, Urmi Trivedi, Michael Kroiher, Michael A.S. Thorne, Einhard Schierenberg, Thomas Wiehe, Mark Blaxter

**Affiliations:** 1 CLOE, Department for Biosciences, University College London, London, UK; 2 Zoologisches Institut, Universität zu Köln, 50674 Köln, Germany; 3 INRA, Université Côte d‘Azur, CNRS, ISA, France; 4 Maynooth University Department of Biology, National University of Ireland Maynooth, Maynooth, Co. Kildare, Ireland; 5 School of Biological Sciences, Queen's University Belfast, Belfast, UK; 6 Irish Centre for High-End Computing, Tower Building, Trinity Technology & Enterprise Campus, Grand Canal Quay, Dublin D02 HP83, Ireland; 7 Megazyme, Bray Business Park, Bray, Co. Wicklow A98 YV29, Ireland; 8 Unidad Académica de Ciencias Biológicas, Laboratorio de Colecciones Biológicas y Sistemática Molecular, Universidad Autónoma de Zacatecas, Zacatecas, México; 9 Institute of Evolutionary Biology, The University of Edinburgh, Edinburgh EH9 3FL, UK; 10 Edinburgh Genomics, School of Biological Sciences, University of Edinburgh, Edinburgh EH9 3FL, UK; 11 British Antarctic Survey, Natural Environment Research Council, High Cross, Madingley Road, Cambridge CB3 0ET, UK; 12 Institut für Genetik, Universität zu Köln, 50674 Köln, Germany

**Keywords:** Evolutionary Biology, Phylogenetics, Transcriptomics

## Abstract

Most animal species reproduce sexually and fully parthenogenetic lineages are usually short lived in evolution. Still, parthenogenesis may be advantageous as it avoids the cost of sex and permits colonization by single individuals. Panagrolaimid nematodes have colonized environments ranging from arid deserts to Arctic and Antarctic biomes. Many are obligatory meiotic parthenogens, and most have cryptobiotic abilities, being able to survive repeated cycles of complete desiccation and freezing. To identify systems that may contribute to these striking abilities, we sequenced and compared the genomes and transcriptomes of parthenogenetic and outcrossing panagrolaimid species, including cryptobionts and non-cryptobionts. The parthenogens are triploids, most likely originating through hybridization. Adaptation to cryptobiosis shaped the genomes of panagrolaimid nematodes and is associated with the expansion of gene families and signatures of selection on genes involved in cryptobiosis. All panagrolaimids have acquired genes through horizontal gene transfer, some of which are likely to contribute to cryptobiosis.

## Highlights

• Parthenogenesis in *Panagrolaimus* nematodes arose once through a hybridization event
• Parthenogenetic *Panagrolaimus* are triploid but retain meiosis after millions of years
• *Panagrolaimus* can be used as a model system to study aspects of animal parthenogenesis
• The genomes of *Panagrolaimus* nematodes are shaped by their adaptation to cryptobiosis

## Introduction

Despite the general prevalence of sexual reproduction in animals, parthenogenesis has evolved in many taxa. Obligatory parthenogenesis, or more strictly the cessation of outcrossing after meiotic recombination, is thought to result in the gradual accumulation of deleterious mutations that cannot be purged independently of all linked loci (Lynch et al., 1993). This would condemn non-recombining parthenogenetic animals to evolutionary dead-ends through Muller ratchet (Felsenstein, 1974) and predicts restriction of these species to terminal phylogenetic branches. The evolutionary scandals of the phylogenetic persistence, and abundant speciation, of some asexual taxa (such as bdelloid rotifers and darwinulid ostracods) may be explained by the recruitment of recombination-like mechanisms (Signorovitch et al., 2015). Up to 38% of species in some arthropod genera may be parthenogenetic (Van Der Kooi et al. 2017), and this mode of reproduction has arisen frequently in nematodes (Denver et al., 2011). Parthenogens have been frequently observed to amplify their genomes through allo- or autopolyploidy, and this may buffer lineages against mutation accumulation in essential genes (Archetti, 2004). Allopolyploid genomes might also enable diversification of gene copies by associating several adapted and diverged genomes in a single hybrid species (Blanc-Mathieu et al., 2017). Comparisons of related sexual and asexual taxa are required to understand the origins and persistence of asexuality and the effects of asexuality on genome evolution.

Asexual reproduction has long been associated with organisms found in extreme or peripheral environments, such as arid deserts where water availability is ephemeral and unpredictable, or polar ecosystems where much of the annual cycle involves temperatures well below zero degree Celsius (Vrijenhoek and Davis Parker, 2009). Because they do not need to find a partner to mate and reproduce, parthenogens can colonize ephemeral or widely spaced niches as single reproductive propagules (Lynch, 1984). Organisms that inhabit extreme environments are often also cryptobionts. Cryptobiosis is the ability of an organism to survive with no evidence of active metabolism under conditions inimical to life. Cryobionts survive freezing, avoiding lethal ice-crystal disruption of biological membranes, whereas anhydrobionts survive loss of effectively all their body water. Cryobionts are frequently also anhydrobiotic, as ice sublimation in very low temperatures can result in freeze-drying. The mechanistic bases of cryptobiosis have been explored in several animal species including *Aphelenchus* nematodes (Reardon et al., 2010), the chironomid insect *Polypedilum vanderplanki* (Gusev et al., 2014), and the tardigrades *Ramazzottius varieornatus* and *Hybsibius exemplaris* (Hashimoto et al., 2016, Yoshida et al., 2017). Intrinsically unstructured proteins and the carbohydrate trehalose have been identified as important molecular components in anhydrobiotic mechanisms. Intrinsically unstructured proteins, such as the late embryogenesis abundant (LEA)-like proteins (Tyson et al., 2012, Gusev et al., 2011), and the tardigrade-specific SAHS, MAHS, and CAHS family proteins (Hashimoto et al., 2016) do not adopt a stable secondary structure in solution and are thought to replace structural water on dehydration or freezing. The polar sugar trehalose is likely to act in a similar way. Some species are able to undergo cryptobiosis rapidly at any stage of development (Hashimoto et al., 2016), whereas others require extensive preconditioning, presumably to activate physiological systems that produce anhydro- or cryo-protectants. Asexuality and cryptobiosis may be mechanistically unlinked, but both traits are advantageous to organisms colonizing extreme habitats.

Many asexual and/or cryptobiotic nematode taxa have been described. Panagrolaimidae are members of Tylenchina, in clade IV (Blaxter et al., 1998) of Nematoda. They include both outcrossing (with male-female) and parthenogenetic (female-only) taxa. Many panagrolaimids are cryptobionts, with both cryobiotic and anhydrobiotic abilities, and are found in extreme environments in polar and temperate regions (Shannon et al., 2005). Although parthenogenetic panagrolaimids are widely distributed around the globe, a single origin of parthenogenesis in the genus *Panagrolaimus* is likely (Lewis et al., 2009). The evolutionary age of the asexual *Panagrolaimus* species is unknown. Some *Panagrolaimus* species are very good cryptobionts, surviving rapid desiccation, and many species are able to survive desiccation at any life cycle stage without pre-conditioning (Shannon et al., 2005). Here we compared the genomes and transcriptomes of parthenogenetic and outcrossing cryptobiotic *Panagrolaimus* species and one *Propanagrolaimus* species. The *Propanagrolaimus* species is not a good cryptobiont. We identified a single, recent origin of parthenogenesis in *Panagrolaimus* species involving triploidization. In contrast, using the same methodology, we found retention of diploidy in two other nematode parthenogens. We identified candidate genes that may be important for cryptobiosis, some of which are likely to have been acquired horizontally from non-metazoan sources.

## Results

### Parthenogenetic *Panagrolaimus* Have Larger Genomes Than Their Sexual Congeners

**Figure 1:**
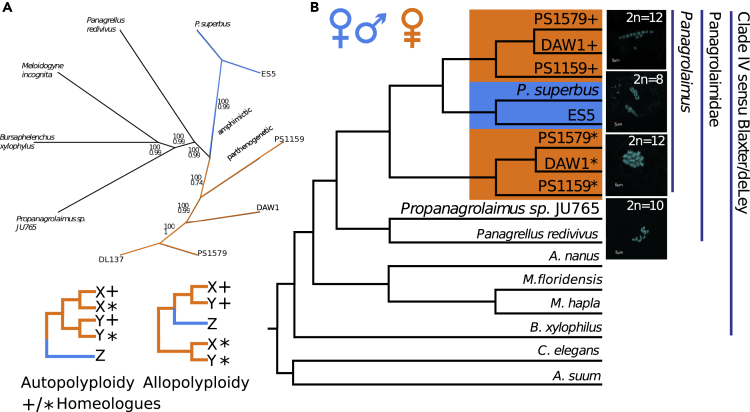
Polyploidy in Parthenogenetic Panagrolaims (A) Phylogenomic analysis of panagrolaimids confirms a single origin of parthenogenesis in the genus. The RAxML tree of the *Panagrolaimus* (including transcriptomic data for PS1579 and DL137), *Panagrellus*, *Propanagrolaimus* is based on CEGMA KOGs, and the topology is congruent with a Phylobayes tree of the same species set. (B) Additional gene copies derive from an independent genome. MUL-trees based gene-tree reconciliation of 15,000 trees from OrthoFinder showing the most parsimonious phylogenetic position for proteins where the parthenogenetic species had multiple copies in a given orthologous group. The + and * symbols designate the different copies per protein in the parthenogenetic species. This topology supports the assumption of polyploid hybrids (allopolyploidy), where the additional proteins in the parthenogens derive from a lineage outside the sampled outcrossing and parthenogenetic species (see tree sketches bottom left for a companions of patterns expected under allopolyploidy and autopolyploidy). All analyzed parthenogenetic species had 12 chromosomes in comparison with 8 in the diploid state of the outcrossing species. The outgroup *Propanagrolaimus* has 2n = 10 chromosomes.

We sequenced and assembled *de novo* the genomes and transcriptomes of five panagrolaimid nematodes (Table 1, Figure 1) using contamination- and heterozygosity-aware methodologies. We reassembled the genome of *Panagrolaimus* sp*.* DAW1, which was highly fragmented and contaminated with a considerable amount of bacterial DNA in the original assembly (Thorne et al., 2014).

**Table 1:** Assembly and Annotation Metrics for the *Panagrolaimus* and *Propanagrolaimus* Genomes Presented in This Study

Species	Span (Mbp)	Ns (k) % of Total	No. Scaffolds (k)	Max. Scaffold Size (k)	N50 Scaffolds	Protein Models (k)	Repeats (%)	GC (%)	BUSCO C + P (%)^a^
*Panagrolaimus superbus*	76	1.095 (1,4)	53.2	29	1,894	24.2	8,4	31,8	96
*P.* sp. ES5	90	1.578 (1,7)	25.2	96.2	6,312	24.8	8	29	94
*P.* sp. PS1159	85	1.702 (2)	17.6	142.9	9,924	27.4	6,8	28,2	94
*P.* sp. PS1579	49	1.185 (2.4)	23.4	24.9	2,935	18 (47.7)^b^	4,2	30,5	95
*P. sp.* DAW1	118	73 (0.06)	39.6	42.2	4,454	31.6	10,3	27,8	68
*Propanagrolaimus* sp. JU765	64	807 (1,3)	13.3	907.5	10,861	24.9	9,1	31,8	94

Data are given for scaffolds >500 bp and contigs >100 bp (scaffolds split at ≥ 10 Ns).

a See Supplement Information for BUSCO output tables.

b The *P*. sp. PS1579 genomic dataset was of low coverage (see main text) and transcriptome (RNA-seq)-based models were used in a complementary purpose.

We independently estimated the genome sizes of strains in culture using Feulgen Image Analysis Densitometry (FIAD) and quantitative PCR (RT-D-PCR). For the outcrossing *Panagrolaimus* isolates diploid genome sizes were estimated to be between 0.14 (*Panagrolaimus* sp. ES5, using FIAD) and 0.18 (*Panagrolaimus superbus*, using RT-D-PCR) pg DNA per nucleus, or 140–180 Mb. For the parthenogens *Panagrolaimus* sp. PS1159 and *Panagrolaimus* sp. PS1579 we obtained FIAD values between 0.23 and 0.27 pg DNA per nucleus (230–270 Mb), i.e., ∼50% larger than the outcrossing strains. The diploid genome size of *Propanagrolaimus* sp. JU765 (previously known as *Panagrolaimus* sp. JU765, see Schiffer et al. (2014) for re-naming) was estimated at 0.13 pg DNA per nucleus (130 Mb).

The spans of the haploid genome assemblies of *P.* sp. ES5 and *P. superbus* (90 and 76 Mb, respectively, Table 1) are congruent with the sizes estimated by densitometry and PCR. Similarly, the haploid genome span of the hermaphrodite outgroup *Prop.* sp*.* JU765 was 64 Mb, in line with the FIAD estimate. The correspondence between assembly span and measured nuclear DNA mass was less clear-cut in the parthenogens. The assembly span of *P.* sp. PS1159 was only 85 Mb, similar to that of the haploid genomes of the sexual species but one-third of the physical measurement. The initial assembly span for *P. sp.* DAW1 was 175 Mb. After cleaning and removal of some duplicate contigs stemming from heterozygosity the final scaffolded assembly measured 118 Mb. The difference between estimated genome sizes and assembly sizes for the parthenogens would suggest the presence of genome copies not separated during the assembly.

### Parthenogenetic *Panagrolaimus* Species Are Likely Triploid

We karyotyped 15 *Panagrolaimus* species and found that all the outcrossing species had a total set of 8 chromosomes (suggesting *2n=8*), whereas all the parthenogenetic species had 12 chromosomes in total (suggesting *xn=12*) (Figure 1, Figure S1). This could correspond to a shift in haploid chromosome number from *n=4* to *n=6*, or triploidy (where *n=4* is maintained but somatic nuclei are *3n*). Observation of polar bodies in the parthenogenetic species shows that meiosis is present (Figure S1).

In polyploid systems, extra copies of most genomic regions will exist. However, if sequence similarity between these copies is high, the genome assembly process will collapse them and only one copy will be present in the predicted gene and protein set. In evolutionarily old parthenogens that lack recombination, independent accumulation of mutation in alleles can lead to sequence divergence (Welch and Meselson, 2000) and thus representation of both alleles in the genome assembly. Similarly, in allopolyploids homeologs may be divergent enough to be independently assembled (Blanc-Mathieu et al., 2017, Glover et al., 2016, Krasileva et al., 2013).

The assembled panagrolaimid genomes showed high completeness, comparable with other *de novo* sequenced genomes for the eukaryotic gene set in the BUSCO pipeline (Simão et al., 2015) (Table 1, Data S1). We performed gene-finding on the new and reassembled genomes and predicted proteins from the transcriptomes of *Panagrolaimus* sp. DL137 and *P.* sp. PS1579. We retrieved domain annotations for 77%–88% of the predicted coding genes in each species. These were clustered with OrthoFinder and analyzed with kinfin (Laetsch and Blaxter, 2017). We included proteomes from *Propangrolaimus* sp. JU765 (this work) and other Tylenchina (Clade IV) nematodes: *Panagrellus redivivus* (Srinivasan et al., 2013), *Meloidogyne hapla* (Opperman et al., 2008), *Acrobeloides nanus* (Schiffer et al., 2018), and *Bursaphelenchus xylophilus* (Kikuchi et al., 2011).

A previous phylogenetic study using neighbor joining and maximum parsimony methods based on fragments of the two rRNA genes (18S, 28S) and one mitochondrial gene (ND5) had indicated a single origin of parthenogenesis in *Panagrolaimus* (Lewis et al., 2009). Drastically extending the set of analyzed genes to all CEGMA KOGs and using the superior maximum likelihood and Bayesian methods to infer phylogenetic trees we were able to confirm a single origin of the parthenogens (Figure 1). We found an excess of protein copies in each of the parthenogenetic *Panagrolaimus* species in over 1,600 clusters of orthologs (orthogroups). Using the gene-tree reconciliation approach implemented in GRAMPA (Gregg et al., 2017) on FastME (Lefort et al., 2015) trees built from these orthogroups, we tested if auto- or allopolyploidy was the source of an extra set of proteins in the parthenogens (Figure 1). If the extra copies in the parthenogens resulted from autopolyploidy they should be more similar to each other than to orthologous copies in the outcrossing outgroup. The most parsimonious placement for these extra proteins was as basal to a clade containing both parthenogenetic and sexual species (Figure 1). It is thus unlikely that the extra copies arose by whole genome duplication within the parthenogenetic lineage (autopolyploidy). The tree topology rather supports an allopolyploid (hybrid) origin for these extra gene copies in the parthenogens.

**Figure 2:**
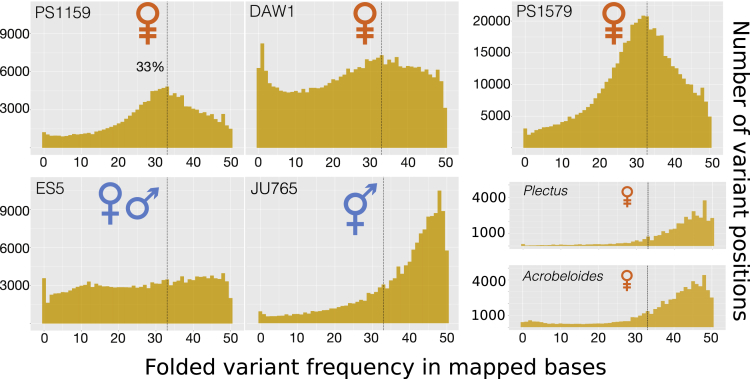
Ploidy and Heterozygosity Estimates Based on Distribution of Variant Frequencies Mapping RNA-seq reads to predicted coding sequences of the respective genomes and counting the occurrence of variant frequencies, we observe a peak of variants at a frequency of 1/3 for the parthenogenetic species. Distribution of variant frequencies in the homozygous genome of the outcrossing diploid ES5 does not show any clear peak, whereas the hermaphrodite JU765 shows the 1/2 frequency peak expected under diploid heterozygosity. Patterns observed in the parthenogens *Acrobeloides nanus* and *Plectus sambesii* are similar to those in JU765, thus suggesting a diploid heterozygous structure too.

To further assess ploidy levels in the parthenogens, we also examined read coverage of variants in *P.* sp. PS1159 and presence of homeologous loci in *P. sp.* DAW1. We mapped RNA sequencing (RNA-seq) data on the predicted coding sequences (CDS) and after variant calling plotted the distribution of allele (variants) frequencies. For the diploid outcrossing *P.* sp. ES5 sequenced from a homozygous population, we observed a flat distribution of allele frequencies, with no evident peak, which is consistent with its homozygous nature (Figure 2). A similar pattern was observed in *P. superbus* (not shown). In the hermaphrodite *P.* sp JU765, distribution of variant frequencies showed a mode of variants present at 50%:50%, consistent with a diploid heterozygous genome. However, in the parthenogenetic taxa we observed a peak in allele frequencies at ∼33%, consistent with the presence of three gene copies at these sites (Figure 2). Using a two-sample Kolmogorov-Smirnov test we confirmed that the allele frequency spectra of the parthenogenetic and outcrossing *Panagrolaimus* species, as well as the parthenogenetic *Panagrolaimus* and androdioecious (hermaphrodite) *Propanagrolaimus*, belong to different distributions (see Table S1). Mapping of genomic reads to the CDS, repeat-masked genomes, and un-masked genomes resulted in similar allele frequency profiles (data not shown). These data, together with observed chromosome numbers support triploidy in the parthenogenetic *Panagrolaimus* species.

We explored the genomes of other parthenogenetic nematodes for similar signals of polyploidy. We analyzed the genome and transcriptome of the Tylenchine (Clade IV) species *Acrobeloides nanus* (Schiffer et al., 2018) and the genome of the Chromadorid (Clade C) species *Plectus sambesii* (Rosic et al., 2018). We found that the allele frequency spectra of both species resembled that of the heterozygous (selfing) hermaphrodite *P.* sp. JU765 (Figure 2). These findings argue for non-polyploid status of these parthenogens.

### Parthenogenesis in *Panagrolaimus* Is 1.3–8.5 Million Years Old

**Figure 3:**
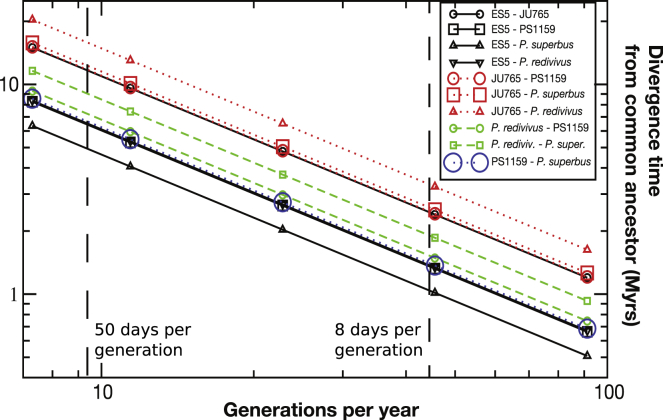
Panagrolaimus Species Diversification Age Using our own generation time measures, genome-wide divergence calculated with Andi, and previous data for *Caenorhabditis* species as reference, we calculated possible age of divergence between the different species and the origin of parthenogenesis in *Panagrolaimus*. Eight days per generation was measured in the laboratory and would indicate an approximate age of 1.3 mya for the PS1159/*P. superbus* split (see Table 3). Fifty days per generation would be a realistic estimate for species living in harsh conditions and would suggest a more ancient origin.

Because they lack the advantages provided by sexual reproduction (e.g., allele shuffling, efficiency of selection), most parthenogenetic species are expected to be recent and to be evolutionary dead ends not giving rise to daughter taxa. To estimate divergence time between the parthenogenetic and outcrossing panagrolaimids, we calculated pairwise divergence of entire genome sequences with ANDI (Haubold et al., 2015). As calibration we used available divergence time estimates for *Caenorhabditis* species (Cutter, 2008), corrected by generation time measurements in *Panagrolaimus* and *Propanagrolaimus*. The outcrossing species *P. superbus* and *P.* sp. ES5 were the most recently separated species (Table 2, Figure 3, see phylogeny in Figure 1). Assuming an average of 8 days per generation, the split between parthenogenetic and outcrossing *Panagrolaimus* species is estimated to have occurred 1.3–1.4 Mya. In natural conditions, it is possible that the *Panagrolaimus* species spend most of the time as non-metabolic cryptobionts. Applying a very conservative generation time of 50 days, the split would have occurred 8.0 to 8.5 Mya.

**Table 2:** Pairwise Divergence Time Estimates in *Panagrolaimus* spp. Based on 8 Days per Generation (Measured by Us) and 50 Days per Generation as Lower Bound

Species A	Repro	Species B	Repro	Myr (8 days/gen)	Myr (50 days/gen)
*P. superbus*	A	ES5	A	1	3.2
PS1159	P	ES5	A	1.33	8.25
PS1159	P	*P. superbus*	A	1.37	8.55
ES5	A	*P. redivivus*	A	1.35	8.35
PS1159	P	*P. redivivus*	A	1.45	9.3
*P. superbus*	A	*P. redivivus*	A	1.88	11.6
ES5	A	JU765	H	2.4	14.95
PS1159	P	JU765	H	2.4	15.1
*P. superbus*	A	JU765	H	2.5	15.8

Calibrated with divergence times calculated for *Caenorhabditis* spp. by Cutter (2008). Mode of reproduction: **A**mphimictic, **H**ermaphroditic, **P**arthenogenetic. See Supplement Information.

### Gene Copies of Potentially Lethal Genes Are less Likely Divergent in the Triploid Hybrids

The evolution of parthenogenesis through hybridization is rapid. Newly formed hybrids may undergo genome and transcriptome shock, as co-adapted complexes can be disrupted by the new ploidy and interacting partners that have accumulated differences through drift are brought into association. Such maladapted interactions need to be eliminated or moderated, and, in the case of parthenogens especially, the machineries of sex determination, fertilization/oocyte activation, and development are likely to undergo accelerated evolution. In parthenogens, the cost of producing males, which play no part in species fitness, is particularly high.

No males have been observed by us in any of the parthenogenetic *Panagrolaimus* species, even under stress conditions (e.g., prolonged culturing at elevated temperatures or after reviving cultures from cryptobiosis). We therefore examined panagrolaimid orthologues of genes involved in several genetic regulatory networks (GRNs) implicated in reproduction and sex determination that have been experimentally defined in *Caenorhabditis elegans*. Using stringent orthology analysis using OrthoMCL (Li et al., 2003) followed by validation with OrthoInspector (Linard et al., 2015) we identified changes consequent to the evolution of polyploidy and loss of sex (see Supplemental Information). Surprisingly, we found that many *C. elegans* genes acting in important developmental processes were absent not only from all the panagrolaimids tested, but also from other Tylenchina species. We were also unable to detect these genes through synteny analysis (see Supplemental Information), an approach previously successfully applied between *Caenorhabditis* species (Kuwabara and Shah, 1994, Streit et al., 1999, Haag et al., 2002). For example, in the sex determination GRN, Tylenchina nematodes lacked orthologs of the *C. elegans* master regulator *xol-1*, as well as *fem-1*, *fem-3*, *her-1*, and other genes. Similarly, in the endoderm- and mesoderm-forming GRN, tylenchines lacked *med-1*, *med-2*, *end-1*, *end-3*, and *spn-4*, among others. A similar pattern was observed for genes acting in spermatogenesis, early axis definition, DNA repair, and oogenesis. This core divergence from the systems as defined in *C. elegans* makes it difficult to identify changes associated with divergence in reproductive mode and ploidy.

In comparison with their outcrossing relatives, the parthenogenetic *Panagrolaimus* species had an excess of genes, many of which were duplicates. The most common fate of duplicated genes is loss through accumulation of deleterious mutations or other mechanisms. This fate is especially likely for loci that are sensitive to dosage or have strong phenotypes when mutated. We identified clusters of orthologs that contained exactly one protein in *C. elegans*, the two outcrossing, and all three parthenogenetic *Panagrolaimus* species (1:1: …:1 orthologs), as well as clusters where the parthenogenetic *P.* sp. PS1159 had more proteins than the obligate outcrossing *P.* sp. ES5 (parthenogen-excess clusters). Using the database of *C. elegans* RNAi phenotypes on wormbase.org (https://wormbase.org; Lee et al., 2018) we retrieved phenotypes for 172 of the 703 single-copy one-to-one orthologues and 695 phenotypes for the 1,618 parthenogen-excess clusters. A chi-square test confirmed (p value = 0.0015) that the proportion of lethal phenotypes (53%) in the 1:1 orthologues was higher and significantly different from the proportion (40%) in parthenogen-excess clusters. This result is in accordance with the expectation that essential genes will be less tolerant to changes in dosage.

### *Panagrolaimid* Species Have Acquired Genes through Horizontal Transfer and These May Impact Cryptobiosis

**Figure 4:**
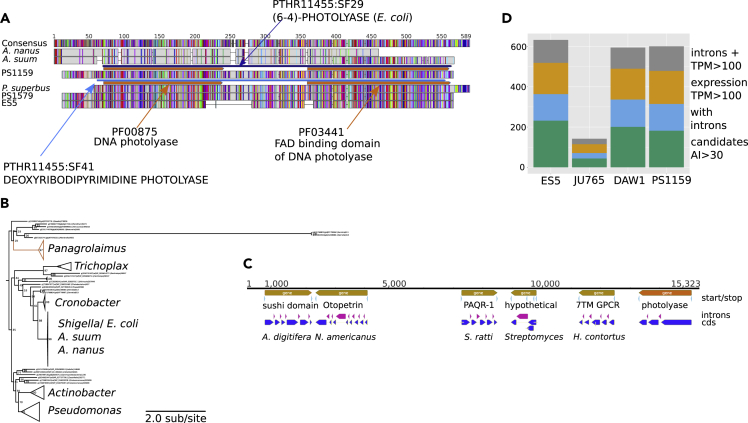
Validating HGT Candidates (A) Protein alignment of putative HGT photolyases found in *Panagrolaimus*, *A. suum*, and *A. nanus* with bacterial homologues. Brown horizontal bars denote Pfam domains, whereas blue colored bars are PANTHER classification (both InterProScan derived). The alignment shows that the *A. nanus* (clade IV) and *A. suum* (clade III) sequences are similar but different from the ones in the three panagrolaims. The PANTHER classification indicates that the former two are very similar to *E. coli* photolyases in these very divergent nematode species, making a contamination originating from bacteria used in worm culturing likely. (B) Phylogenetic analysis of photolyases from *Panagrolaimus*, *A. suum*, and *A. nanus* and bacteria. The analysis further supports that the *A. suum* and *A. nanus* proteins are *E. coli* contamination, whereas the *Panagrolaimus* proteins are distinct acquisitions from other taxa. (C) Genomic integration of HGT photolyase. The photolyase locus in *P. sp.* PS1159 was found on the same contig as *bona fide* nematode proteins, further substantiating the conclusions that the gene was horizontally acquired and fully integrated into the nematode genome. (D) Classification of HGT candidates in Panagrolaimidae. A stacked histogram of AI>30 HGT candidates for the different species classified by their features. Those with introns and expression levels of TPM>100 can be regarded as the most reliable.

We identified potential horizontal gene transfers (HGTs) using the Alien Index (AI) approach as implemented in Alienness (Rancurel et al., 2017). We identified from 22 (*P. redivivus*) to 232 (*P.* sp. ES5) likely HGT candidates from non-metazoan donors into the genomes of panagrolaimids (Table 3, Data S4, S5, S6, S7, S8, and S9). We used a series of additional screens to confirm HGT and rule out contamination, including a check for spliceosomal introns, codon usage, presence of evolutionarily conserved metazoan genes on the same contigs, and transcriptional support from RNA-seq data (Figure 4). The inferred source taxa for the majority of high-confidence HGT candidates were Bacteria followed by Fungi in all *Panagrolaimus* species except *P. sp.* DAW1 (in which Fungi were the most frequent, followed by Bacteria).

**Table 3:** Protein Numbers, HGT Candidates, and High-Confidence HGT Candidates in the *Panagrolaimus* Genomes and Outgroups

Species	Total # Proteins	Possible HGT Candidates (AI >0)	Candidates Identified as Contaminants^a^	# HGT Candidates with AI >30	High-Confidence HGT Candidates^b^					
					# on Same Contig with Other Gene	# with Neighbors Hitting Metazoa	# with introns	# with Expression >10/100/1000 TPM in RNA-seq	# with Pfam Domains (Total Number of Pfam Domains)	Distinct Pfam Domains
*Panagrolaimus superbus*^b^	24,160	528	20	88	NA	NA	NA	NA	70 (91)	56
*Panagrolaimus* sp. ES5	24,756	975	141	232	215	73	132	185/155/97	183 (429)	136
*Panagrolaimus* sp. PS1159	27,350	899	19	182	159	98	133	174/164/112	141 (268)	93
*Panagrolaimus* sp. DAW1	31,646	1,177	18	201	194	84	136	178/153/84	131(172)	62
*Propanagrolaimus* sp. JU765	24,878	530	39	44	42	25	27	44/44/36	34 (100)	23
*Panagrellus redivivus*	26,372	516	12	22	21	21	16	NA	14 (18)	12

a AI>0 and >70% id to non-metazoan protein.

b Owing to the shortness of the contigs and a low-coverage 454 transcriptome we refrained from calculating high-confidence candidates for *P. superbus*.

**Figure 5:**
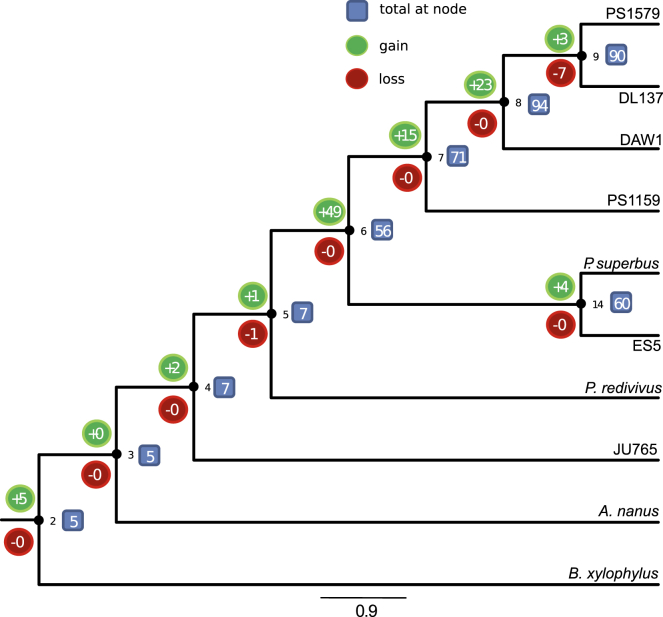
Reconstruction of the Timing of Acquisition and Loss of HGT Candidates The distribution of HGT candidates across these species was mapped to a phylogeny of Panagrolaimidiae and two outgroups and ancestral states deduced with a parsimony approach. Internal nodes are numbered (2–9 and 14). The number of gene families acquired via HGT/lost at each node are represented in green/red bubbles, respectively. The total number of gene families acquired via HGT present at each node is indicated within blue boxes.

We identified 110 OrthoMCL groups that contained candidate HGT from at least two *Panagrolaimus* species. These genes have most likely been acquired horizontally in a common ancestor fo these species (multiple independent acquisitions is less parsimonious). We reconstructed the relative timing of acquisition via HGT for these 110 orthogroups using parsimony (Methods, Figure 5). Most (102; 93%) HGT events were predicted to have taken place in an ancestor of two or more *Panagrolaimus* species. Interestingly, the highest number of acquisitions (49 events) took place in the lineage leading to the last common ancestor of all the cryptobiotic *Panagrolaimus* species. Only seven HGT orthogroups were present in the last common ancestor of all eight panagrolaimid species analyzed. These seven ancestral HGT orthogroups plus the 49 novel acquisitions suggest that at least 56 independently acquired HGT loci were present in the common ancestor of the cryptobiotic *Panagrolaimus* species (Figure 5).

The 56 HGT orthogroups present in the last common cryptobiont ancestor contained a total of 910 proteins, annotated with 192 different Pfam domains (Table 3). Only three Pfam domains were conserved in the candidate HGT sets of all six *Panagrolaimus* species and all corresponded to enzymatic functions (alcohol dehydrogenase GroES-like domain, pyridine nucleotide-disulfide oxidoreductase, and zinc-binding dehydrogenase) (Data S3). HGTs conserved between *P. superbus*, *P.* sp. ES5, *P.* sp. DAW1, and *P.* sp. PS1159 were annotated with thirty conserved Pfam domains that included GH43, GH28, and GH32 glycoside hydrolases, previously identified as loci acquired by HGT in plant-parasitic nematodes (Rancurel et al., 2017) (Haegeman et al., 2011). We found that 47 Pfam domains functionally connected to cryptobiosis were annotated in proteins putatively acquired via HGT, 19 acquired by the last common ancestor of *Panagrolaimus* and 5 only found in the parthenogenetic species. Functions associated with these domains included peptidase, diapausin, killer toxins of the Kp4 family, LURP-one-related proteins, flavin-binding monooxygenase-like protein, caleosin, and photolyase. Proteins with these functions fulfill various roles in defense against pathogens and the removal of xenobiotics The photolyase (Figure 4) was of particular interest, since photolyases are able to repair UV-induced damage in DNA and have been lost in several branches of Metazoa (Lucas-Lledó and Lynch, 2009). Desiccation can induce DNA damage and thus genome repair is important for desiccating organisms (Jönsson and Schill, 2007). The caleosin found in *Panagrolaimus* is the first report to date of animal caleosins. Caleosins have previously been described only in multicellular plants, green algae, and fungi (Partridge and Murphy, 2009). Plant caleosins expressed in non-seed tissues are responsive to a range of environmental stresses, particularly salinity and dehydration (Blée et al., 2014).

### *Panagrolaimus* Genomes Are Shaped by Gene Families Potentially Implicated in Cryptobiosis

Adaptation to novel (extreme) habitats can also involve expansion and diversification of gene families. We analyzed gene family expansions in the cryptobiotic panagrolaimid species compared with related taxa. Forty-three Pfam domains were overrepresented in the cryptobiotic *Panagrolaimus* species compared with an outgroup containing *Prop*. sp. JU765 and *Panagrellus redivivus* (which are not cryptobionts), after Benjamini-Hochberg correction, and 123 Pfam domains were overrepresented in Panagrolaimidae (*Panagrolaimus, Propanagrolaimus,* and *Panagrellus*) compared with the remaining taxa (Figure S2).

As an approach complementary to overrepresentation tests, we programmed a support vector machine (SVM) classifier based on Pfam domain annotations to distinguish cryptobiotic *Panagrolaimus* from *Propanagrolaimus* plus *Panagrellus*. In the *Panagrolaimus versus Panagrellus*/*Propanagrolaimus* SVM analyses, 64 domains were frequently associated with successful classification, of which 13 were common to the SVM and the Pfam enrichment Null hypothesis significance testing (NHST) approaches (Figure S2). These 13 domain annotations included several that may play a role in cryptobiosis, including Hsp70 protein domains, serine protease inhibitors, ubiquitin family domains, lipase domains, and enoyl-reductase domains. Annotations found to be overrepresented only in the NHST analyses were proteolysis (subtilase family and matrixin domains) and maintenance of genome integrity (PIF1-like helicase, deoxyribonuclease II, linker histone H1 and H5 family).

### Repeat and Transposon Content and Functional Analysis of the *Pangrolaimus* Gene Space

For additional information on Panagrolaimid genome assemblies as well as pertinent comparative features, please refer to the Supplementary Results and Discussion section within the Supplemental Information file.

## Discussion

Understanding how parthenogenetic animals evolve and whether they persist through evolutionary time is critical to answering the fundamental biological question of why recombinational sex is near-ubiquitous in animals. How animal species adapt to environmental challenges is similarly of fundamental interest. It has been proposed that both traits are linked in geographical parthenogenesis (Vrijenhoek and Davis Parker, 2009). Many parthenogenetic nematode species have been shown to be polyploids of hybrid origin (Lunt, 2008, Castagnone-Sereno and Danchin, 2014, Lunt et al., 2014, Hiraki et al., 2017, Kraus et al., 2017, Blanc-Mathieu et al., 2017). In animals, it is assumed that polyploidy, especially when uneven, leads to non-viability and that living hybrids may suffer a reduced fitness (Burke and Arnold, 2001). Alternatively, polyploid parthenogens might have a fitness advantage in that they are buffered against Muller ratchet (Lynch, 1984). Hybridization between species frequently leads to aberrant meiosis in animals, and a possible escape from this dilemma could be parthenogenesis (Avise, 2008). In such parthenogens, incorporation of a round of chromosomal endoreplication is a possible route to maintain meiotic triploidy through oogenesis (Lutes et al., 2010).

We have analyzed the genomes and transcriptomes of parthenogenetic and outcrossing *Panagrolaimus* nematodes, which, in contrast to closely related *Propanagrolaimus* and *Panagrellus*, are capable of surviving under prolonged drying (anhydrobiosis) and freezing (cryobiosis). Our combined karyotypic and molecular data indicate that the parthenogenetic *Panagrolaimus* are triploid hybrids originating from a single event. All analyzed parthenogens have a karyotype of n = 12 (compared with diploid n = 8 in amphimicts), an excess of duplicated genes that share a single phylogenetic origin, and variant frequency spectra compatible with triploidy. Triploidy could have arisen by autopolyploidy (duplication of one set of ancestral chromosomes, for example, by fertilization of a diploid oocyte by a haploid sperm from the same species) or allopolyploidy (where the additional haploid complement comes from a distinct lineage). These contrasting origins predict contrasting patterns of divergence between the three copies of each locus. In parthenogenetic *Panagrolaimus* we find phylogenetic evidence for deep divergence of the extra copies and probable acquisition from a donor species positioned between the *Propanagrolaimus* and *Panagrolaimus* species present in our analysis. These results support allotriploidization. Analyzing two further parthenogenetic nematodes, the tylenchine cephalobe *A. nanus*, and the distantly related plectid *P. sambesii*, we found contrasting signal. Both species appear to be diploid. It is thus likely that they became parthenogenetic through a different route than the *Panagrolaimus* species. In the Rhabditine (Clade V) genus *Diploscapter* (the sister genus to *Caenorhabditis*) parthenogenetic species appear to show allelic divergence, but whether parthenogenesis involved hybridization in the genus remains unresolved (Fradin et al., 2017, Hiraki et al., 2017, Kraus et al., 2017). Thus parthenogenesis has arisen independently and repeatedly in Nematoda (Holovachov et al., 2013), offering a rich set of systems in which to investigate the mechanisms and subsequent effects of the loss of sexual reproduction.

Several kinds of genes associated with cryptobiosis have been identified in the panagrolaimid nematodes. Plant embryos express at high levels LEA proteins, natively unstructured peptides that are believed to surround proteins in a “molecular shield” (Chakrabortee et al., 2012). Natively unstructured proteins have been implicated in anhydrobiotic responses in nematodes (*Aphelenchus avenae* [Reardon et al., 2010]), arthropods (*Polypedilum vanderplanki* [Cornette et al., 2010]), and tardigrades (*R. varieornatus* and *H. dujardini* [Horikawa et al., 2013]). Other gene families associated with anhydrobiosis include protein kinases, proteasomal components, ubiqutin, protease inhibitors, proteases, DNA repair enzymes, enzymes with roles in oxidative stress protection, and heat shock proteins (Cornette et al., 2010).

In cryptobiotic tardigrades, in addition to a series of tardigrade-specific natively unstructured protein families, genomic and transcriptomic analysis highlighted the involvement of key stress response pathways in anhydrobiosis. These include horizontally acquired catalases and anhydrobiosis-upregulated superoxide dismutases and glutathione-S transferases, enzymes involved in detoxification and protection against oxyradical damage. DNA damage signaling and repair genes were also associated with anhydrobiosis. Surprisingly, the mTOR signaling pathway was absent in the tardigrades, as were the HIF-1alpha cascade and many genes involved in peroxisomal functions including beta oxidation and other H_2_O_2_ producing pathways. It has been proposed (Hashimoto et al., 2016, Yoshida et al., 2017) that their absence in tardigrades is a mechanism to avoid induction of stress-related apoptosis in cells undergoing anhydrobiosis. In the panagrolaims, however, we found no loss of these pathways. Thus the molecular machinery of desiccation does not follow convergent evolutionary paths in these taxa.

Horizontal gene transfer has emerged as an important process in animal evolution (Danchin, 2016) and has been associated (not un-controversially) with cryptobiosis. We identified about 150 gene families in *Panagrolaimus* and Panagrolaimidae that had signatures of acquisition through HGT, many of which were mapped to the root of the cryptobiotic *Panagrolaimus* species group. These candidate HGT genes have acquired spliceosomal introns, have similar GC content and codon usage to resident *Panagrolaimus* genes, are assembled in the vicinity of bona fide nematode genes, and are expressed as poly(A)+ RNA. They can be regarded as domesticated and functionally integrated into the host genomes. Functional annotation of the putative HGT loci in *Panagrolaimus* suggested that they contribute to anhydrobiotic physiology and feeding. Genes acquired through HGT were also implicated in cryptobiosis in the rotifer *A. vaga* and the tardigrades *R. varieornatus* and *H. dujardini*, and a functional link between anhydrobiosis and HGT has been proposed (Hespeels et al., 2014). However, comparative genomic analyses in rotifers showed that the proportion of genes acquired by HGT was equally high in desiccation-intolerant and -tolerant rotifers (Nowell et al., 2018). The same analysis extended to other protostomes showed no clear link exists between abundance of HGT and desiccation tolerance (ibid).

The level of HGT in panagrolaimid nematodes appears similar to that found in hypsibid tardigrades (Yoshida et al., 2017). Only a small fraction of the total gene number, around 1% or less, is horizontally acquired. Nevertheless, the relative proportion of strongly supported HGT candidates per genome is higher in the cryptobiotic panagrolaimid species in comparison with the non-cryptobiotic outgroups, hinting at a potential connection between the lateral acquisition of genes and cryptobiosis. Whether this is mechanistic (i.e., that taxa that experience drying or freezing are more likely to take up DNA from their environment) or causal (i.e., the HGTs underpin cryptobiosis) is presently not resolvable.

We identified karyotypic signatures of meiosis in the parthenogenetic *Panagrolaimus* species. However, the exact mode of restoration of their triploid status after meiosis remains unresolved. An aberrant tripolar spindle forming during meiosis I contributes to the maintenance of the correct (3n) ploidy in triploid carp (the goldfish *Carassius auratus langsdorfii*) (Yamashita et al., 1993). In parthenogenetic triploid *Poeciliopsis* fish endomitosis precedes meiosis, raising the number of chromosomes to 6n to ensure that a triploid egg is generated at the end of meiosis (Cimino, 1972). Identification of the parthenogenetic mechanism in the *Panagrolaimus* species will need detailed microscopic and cytogenetic analysis.

The parthenogenetic *Panagrolaimus* species appear to be 1.3–8.5 mya old. Although this is older than previously reported cases in vertebrates and most arthropods, this is much younger than the extreme estimates for the bdelloid rotifers and oribatid mites (up to 100 mya) (Neiman et al., 2009). It should be noted that estimating the age of loss of sexual reproduction is not straightforward, and the methods used are completely different for each species. Furthermore, estimates of generation times cannot be easily transferred from the laboratory to nature. For example, the Antarctic species *P. sp.* DAW1 could potentially be restricted to very few generations per year. However, other species are present in temperate regions and thrive in semi-arid (sandy) soils or leaf litter (Shannon et al., 2005) (McGill et al., 2015) where many generations per year are likely. The theory underpinning geographical parthenogenesis holds that parthenogenetic species have an advantage in adapting to new and/or extreme environments. Since we did not observe a strong signal of specific adaptation in the parthenogenetic species (including to the mode of reproduction itself) in comparison with sexual congeners it appears possible that a general-purpose genotype is indeed maintained in these evolutionarily young parthenogens, potentially by combining several differently adapted genomes during hybridization. The same hypothesis was suggested to resolve the surprising parasitic success of root-knot nematodes despite obligatory parthenogenesis (Blanc-Mathieu et al., 2017).

Only a few parthenogenetic species are suitable for genomic and molecular research. We have not yet linked expression of specific *Panagrolaimus* gene families directly to cryptobiosis or parthenogenesis. Our ability to infer function for *Panagrolaimus* genes was limited by phylogenetic distance from the well-annotated *C. elegans* orthologues. *Panagrolaimus* nematodes are easily cultured in the laboratory and accessible to molecular genetic analysis (Schiffer et al., 2014), including RNAi (Reardon et al., 2010). Given the amenability of many cultured nematodes species to genetic manipulation through CRISPR-Cas and other toolkits, we expect these *Panagrolaimus* species will be a valuable platform for further exploration of the genes important for and mechanisms of cryptobiosis.

### Limitations of the Study

Ideally, the genomes presented in this study would have been sequenced with long-read technology and single individuals as source for the input DNA. This might have allowed totally separating and phasing the individual haplotypes of the parthenogenetic and outcrossing species. Unfortunately, sequencing single individuals, although theoretically interesting to analyze the intra-species divergence in the parthenogens, remains technically extremely challenging (and very cost-intensive) with long-read technologies. Hence, one limitation is the fragmented state of the assembled genomes, which precludes most of the synteny analyses.

Although we could assert meiosis in the parthenogenetic Panagrolaimus species, the exact mode of restoration of the triploid status remains unresolved. It has been hypothesized that an aberrant tripolar spindle forming during meiosis I in triploid carps (the goldfish species *Carassius auratus langsdorfii*) is contributing to the maintenance of the correct (3n) ploidy (Yamashita et al., 1993). In triploid parthenogenetic *Poeciliopsis* fish, an endomitosis precedes meiosis. By first raising the number of chromosomes to 6n it is thus ensured that at the end of meiosis a triploid egg is present (Cimino, 1972). Whether similar mechanisms are at play in the *Panagrolaimus* species will need detailed microscopic analysis of the germline in the future.

A further limitation was imposed by the phylogenetic position of the species themselves. Owing to the distant relation to *C. elegans*, a substantial portion of orthologues involved in the earliest steps of development could not be found in the parthenogenetic panagrolaimid species and more generally in the clade IV nematodes. Thus, although we present a tractable model to explore the mechanisms of parthenogenesis in the future, there is still development needed to identify the genes involved.

Finally, unlike the tardigrades analysis, whereby differential expression data for species with different amenability to desiccation was conducted (Yoshida et al., 2017), we were not able to link specific gene families differentially regulated upon cryptobiosis. Our analysis remains a genomic study, yielding no expression data on cryptobiosis. To study which of the identified candidate genes might play an active role during desiccation or freezing, functional molecular studies, like RNAi or CRISPR-mediated gene knock-outs, would be needed.

## Methods

All methods can be found in the accompanying Transparent Methods supplemental file.

## Data and Code Availability

All sequencing libraries are deposited in the SRA under the BioProject PRJNA374706. All genome assemblies are deposited in ENA under PRJEB32708 (*Propanagrolaimus* JU765: ERS3438151; *Panagrolaimus* PS1159: ERS3467865; *Panagrolaimus* ES5_v2 ERS3467866; *Panagrolaimus superbus*: ERS3467867; *Panagrolaimus* PS1579_v2 ERS3468321; *Panagrolaimus* sp. DAW1: ERS3468322). Transcriptome assemblies are deposited in TSA and can be found under the same BioProject as the raw read data (PRJNA374706). All genome assemblies, as well as gene predictions, and corresponding peptide sequences will be made available through WormBaseParasite shortly.

## Supporting Citations

The following reference appears in the Supplemental Information: Zerbino and Birney, 2008.

## Supplemental Information

Document S1. Transparent Methods and Table S1, Related to Figures 1 and 2

Data S1. BUSCO Results, Related to Figure 1 Genome completeness for the newly assembled *Panagrolaimus* and *Propanagrolaimus* species' genomes as determined by the BUSCO pipeline with the eukaryote gene set on gVolante.

Data S2. HGT – GOSlim, Related to Figures 4 and 5 GOSlim annotation for HGT candidates found in the panagrolaimid genomes.

Data S3. HGT – Pfam, Related to Figures 4 and 5 List of Pfam domains found for HGT candidates in panagrolaimid nematodes.

Data S4. HGT AI-PS1159, Related to Figures 4 and 5 Detailed results of the AlienIndex-based primary HGT candidate screen per species in our analysis.

Data S5. HGT AI-JU765, Related to Figures 4 and 5 Detailed results of the AlienIndex-based primary HGT candidate screen per species in our analysis.

Data S6. HGT AI-ES5, Related to Figures 4 and 5 Detailed results of the AlienIndex-based primary HGT candidate screen per species in our analysis.

Data S7. HGT AI-Pdavidi, Related to Figures 4 and 5 Detailed results of the AlienIndex-based primary HGT candidate screen per species in our analysis.

Data S8. HGT AI-Psuperbus, Related to Figures 4 and 5 Detailed results of the AlienIndex-based primary HGT candidate screen per species in our analysis.

Data S9. HGT AI-Predivivus, Related to Figures 4 and 5 Detailed results of the AlienIndex-based primary HGT candidate screen per species in our analysis.

Data S10. Developmental Orthologues, Related to Figure S7 Comparison of groups of orthologues, co-orthologues, and in-paralogues inferred with OrthoMCL and Orthoinspector for genes important in *C. elegans* developmental pathways.

Data S11. dNdS, Related to Figure S9 List of genes possibly under selection and their functional descriptions retrieved from UniProt and Wormbase Genes listed are for comparison between amphimictic and parthenogenetic *Panagrolaimus* species, and for cryptobiotic *Panagrolaimus* species and (non-cryptobiotic) outgroup species.
