# Supplementary material for: Signatures of the Evolution of Parthenogenesis and Cryptobiosis in the Genomes of Panagrolaimid Nematodes
Source: iScience. 2019 Oct 24;21:587–602. doi: 10.1016/j.isci.2019.10.039 (PMC6889759; doi:10.1016/j.isci.2019.10.039)
Supplement: Document S1. Transparent Methods and Table S1, Related to Figures 1 and 2 [file mmc1.pdf]

ISCI, Volume *21*

Supplemental Information

Signatures of the Evolution of Parthenogenesis and Cryptobiosis in the Genomes of Panagrolaimid Nematodes

Philipp H. Schiffer, Etienne G.J. Danchin, Ann M. Burnell, Christopher J. Creevey, Simon Wong, Ilona Dix, Georgina O'Mahony, Bridget A. Culleton, Corinne Rancurel, Gary Stier, Elizabeth A. Martínez-Salazar, Aleksandra Marconi, Urmi Trivedi, Michael Kroiher, Michael A.S. Thorne, Einhard Schierenberg, Thomas Wiehe, and Mark Blaxter

Supplementary Results and Discussion

Repeats

Combining RepeatModeller and RepeatMasker results we found only low proportions of repeats in the *Panagrolaimus* and *Propanagrolaimus* genomes. *P.* sp. ES5 had 7.96%, *P. superbus* 8.43 %, *P.* sp PS1159 6.84% and *Prop.* sp. JU765 9.10% masked bases (we did not evaluate the fragmented *P. sp.* DAW1 genome). These data are similar to the proportions reported for the *P. redivivus* genome (7.1%) (Srinivasan et al. 2013), but considerably less than observed for the *C. elegans* (16.5%) and *C. briggsae* (22.4%) genomes. Using the *k*-mer based approach we identified not only genomic regions that had already been identified and masked by the RepeatModeler and RepeatMasker pipeline, but also additional candidates. This increased the total repeat proportion in the genomes by 1-2% to between 8-10% in total.

	Exact p-value ll KS-statistic	Exact p-value ll KS-statistic
P. sp. PS1159	0.021 ll 16.49	0.015 ll 34.18
P. sp. DAW1	0.0024 ll 36.83	0.036 ll 3.32
	P. sp. ES5	P. sp. JU765

Supplementary Table 1: *Results from Kolmogorov-Smirnov test comparing the variant distributions in triploid parthenogenetic (PS1159, P. sp.* DAW1*) and diploid amphimictic (ES5) and hermaphroditic (JU765) species.*

Transposons

We detected a helitron transposon in a *P. redivivus* small heat shock protein gene. Helitrons are eukaryotic transposons that are predicted to amplify by a rolling-circle mechanism (Kapitonov and Jurka 2001). There may be as many as 560 helitrons in the *C. elegans* genome, located in heterochromatin and in the gene-poor chromosome arms. However, helitrons have been identified in only six *C. elegans* protein coding genes (Eki et al. 2007) and our BLASTp searches failed to find any additional helitron insertions in *C. elegans* coding sequences. By contrast helitron insertions appear to be quite abundant in *Panagrolaimus* protein coding genes. We identified an expansion of helitron Pfams in our InterProsScan analysis (supplementary Fig. 9) and additional BLASTP screens indicated the

presence of 27 independent insertions in PS1159 coding sequences and 49 helitron insertions in *P. superbus* genes.

Developmental Systems

We have previously shown that developmental system drift (DSD) (True and Haag 2001) has modified the gene regulatory networks (GRNs) of endoderm and mesoderm formation between *Panagrolaimus* and *Propanagrolaimus* (Schiffer et al. 2014). Specifically, the expression pattern of the *skn-1* orthologue, a key gene in determining *C. elegans* cell fates, changes between these genera: *skn-1* is expressed during germline specification in *Panagrolaimus*, but not *Propanagrolaimus* (Schiffer et al. 2014). In the current study, we enhanced our analyses by combining the stringent clustering of OrthoMCL with OrthoInspector, which is able to detect more remotely-connected paralogues. For example, in the endoderm/mesoderm induction pathway an *elt-2* orthologue (which is required in *C. elegans* for initiating and maintaining terminal differentiation of the intestine) was not identified in any species outside *C. elegans* by OrthoMCL, but is found by OrthoInspector. However, we found that many genes from the endoderm and mesoderm differentiation pathways in *C. elegans* are missing in all tylenchine (Clade IV) species (supplementary fig. 7) even when using OrthoInspector. The missing genes take part in intermediate switches in the endoderm and mesoderm GRNs. Panagrolaimidae and other tylenchine species are missing not only the *med* and *end* genes, but also have no orthologues of *tbx-35* (which encodes a T-box transcription factor) and *ceh-51* (encoding a homeodomain transcription factor), all of which act downstream of *skn-1* (Maduro and Rothman 2002).

The sex determination pathway has special relevance for the evolution of parthenogenesis, as it is expected that parthenogens will rapidly lose male function to avoid the cost of male production in the absence of the benefit of recombination. We found that many components of the *C. elegans* sex determination pathway were absent either from the panagrolaimids, or from most of the other tylenchine species analysed (Supplementary Excel file Developmental Orthologues). Again, we found a difference between OrthoMCL and Orthoinspector. While neither detected orthologues of the F-Box protein *fog-2* in any of the tylenchine species (or *A. suum*), only OrthoInspector identified the huge number of F-Box in-paralogues known to be present in *C. elegans* (Schiffer et al. 2016). The global picture for the regulation of the panagrolaimid sex determination pathway thus resembled that of the endoderm/mesoderm GRN with upstream (*sex-1*) and downstream players (*fem-2*, *tra-1*) conserved, while intermediate switches were missing (e.g. *her-1*, *tra-2)* (Supplementary Excel file Developmental Orthologues). We found no orthologues of *sdc-*

genes, which act in *C. elegans* dosage compensation, in the tylenchine species. We did find orthologues of *mab-3* (*Drosophila* Doublesex) and related proteins *dmd-4*, *dmd-5*, which are important for male formation in *C. elegans.* Thus, some genes in the canonical sex determination pathway appear to be conserved and could be targets for molecular knock-out studies to investigate the pattern of loss of residual male function in the parthenogenetic taxa.

Sperm function is expected to degrade in parthenogens. *C. elegans* and most other nematode sperm utilize a distinct, non-flagellar motility system involving members of the Major Sperm Protein family (MSP). We identified expressed MSP genes in the *Panagrolaimus* species, including the parthenogens. *C. elegans spe-41* encodes a calcium channel which is crucial for fertilisation in *C. elegans* (Xu and Sternberg 2003), and calcium channels are generally important for fertilisation in animals (Stricker 1999). We found an orthologue of *spe-41* expressed in the parthenogenetic species. We hypothesis that in a hybrid system, and even more so in a polyploid one, imperfect dosage compensation and chromatin-based silencing could lead to expression 'leakage' of 'male-genes', which could then act in the induction of development. It has however been shown that *C. elegans* MSPs can have a role in somatic cells (Schmitz et al. 2007; Schwartz et al. 2012). Thus, similar to the observation of MSP expression in parthenogenetic *A. nanus* and *D. coronatus* (Heger et al. 2010) our finding does not necessarily imply a role of this gene in the reproduction of parthenogenetic panagrolaims. Equally, it is possible that *spe-41* could have a so far undiscovered role in other than sperm cells. It will thus be necessary to conduct differential single cell-transcriptomics analyses and gene expression visualisation studies in the parthenogenetic and closely related male-female species to study the role of these and other genes in asexual reproduction.

DNA repair and parthenogenesis

Efficient DNA repair is particularly crucial for anhydrobiotic species, since exposure to frequent desiccation can lead to DNA strand breaks (Hespeels et al. 2014), and could be equally important under parthenogenesis to avoid mutational meltdown by Muller's ratchet (Muller 1964; Lynch et al. 1993). We identified components of the *C. elegans* DNA repair system in the tylenchine species, and contrasted amphimictic and parthenogenetic *Panagrolaimus* species. We found that most loci were either universally present or absent across Tylenchina (Supplementary Excel file Developmental Orthologues). One exception is *mlh-1*, involved in mismatch repair, which is apparently absent in the parthenogenetic

Panagrolaimus species, but present in the amphimictic representatives and in other tylenchines. To explore and independently validate this pattern of absence, we used figmop (Curran et al. 2014) to build a gene model based on motifs in the *C. elegans* gene and screened the *P.* sp. PS1159 genome. We found one candidate in this genome which was paralogous to the orthologues of *mlh-1* in *P.* sp. ES5, *P. superbus*, and the outgroup. We identified a *Panagrolaimus*-specific region at the N-terminal end of the protein, as well as residues specific to the parthenogenetic species. A splits network (not shown) constructed for the proteins also indicates *Panagrolaimus*-specific divergence. Thus, while the general pattern of presence or absence for orthologues in comparison to the model organism does not allow for inferences about loss or gain of function in the panagrolaims, changes in single genes identifiable in our dataset open avenues for future studies into the molecular underpinnings of parthenogenesis.

Genomic response to cryptobiosis

We found several gene families that could play a role in survival under cryptobiosis to be inflated in number in the *Panagrolaimus* species (also see main text for enrichment results). In particular, small Heat Shock Proteins (supplementary figs. 3, 8), as well as Ubiquitin family genes, BTB/POZ domain containing genes, HSP70 genes, C-type lectins, Piwi domain containing genes, and DEAD/DEAH box containing genes (supplementary fig. 8). Among this group of inflated gene families the HSP70s are important in the protein folding machinery and stress response mechanisms. Similarly, ubiquitin family genes and small Heat Shock proteins, which have been reported expressed in desiccation-tolerant *Aphelenchus* avenae nematodes (Karim et al. 2009), could play a role in response to cryptobiosis. Their inflation in number in comparison to other clade IV species, especially the *Propanagrolaimus* and *Panagrellus* species are thus promising candidates for an involvement in the adaptation to survival under extreme conditions in *Panagrolaimus*.

Present in all domains of life, C-type lectins are a diverse group of proteins involved in protein-protein, protein-lipid, or protein-nucleic acid interactions. In fish, type II antifreeze proteins are derived from C-type lectins (see (Davies 2014)), although what role they play in nematodes specifically in relation to cryptobiotic function is unclear. However, *P sp. DAW1* show an increased transcription of a C-type lectin gene when the nematode is undergoing intracellular freezing (Thorne et al. 2017), suggesting the importance of the gene in the process.

Loci under positive selection in *Panagrolaimus*

We identified genes that could be under positive selection by comparing dN/dS ratios in species with different reproductive modes and comparing cryptobiotic with non-cryptobiotic panagrolaims (supplementary fig. 9). Homologues of the human DNA polymerase zeta (REV3L) were identifed as being under positive selection in parthenogenetic compared to obligate outcrossing *Panagrolaimus*. These might act in DNA repair, maintaining genome integrity in the nematodes. An orthologue of *C. elegans denn-4*, a likely GDP/GTP exchange factor in MAPK pathways, was also possibly under positive selection the parthenogenetic *Panagrolaimus*. Comparing *Panagrolaimus* to other taxa, orthologues of *C. elegans fcd-2*, which acts on cross-linked DNA and DNA damage repair (Vermeulen 2015), had a dN/dS >1, as did helicases similar to *C. elegans* WBGene00010061, an orthologue of human TTF2 (transcription termination factor 2). The TTF2 orthologue will be of interest for follow-up studies, as the human gene is thought to be involved in DNA repair (Hara et al. 1999). In comparison to the parthenogenetic species *fcd-2* had a signature of selection in the sexual species. Similarly, the *Panagrolaimus* orthologue of the *C. elegans* fatty acid and retinol-binding protein *far-6* (Garofalo et al. 2003) appeared to be under positive selection. InterProScan and GO annotation of loci under selection showed significant enrichment of functions such as carbohydrate transport and metabolism, cell wall/membrane/envelope biogenesis, and replication, recombination and repair (supplementary fig. 10, Supplementary Excel file dNdS). Loci contributing to "replication, recombination and repair" annotations include several connected to DNA integrity and processing of nucleic acids (supplementary fig. 9).

Transparent Methods

Nematode strains and culture

Nematodes were acquired from collaborators or through sampling by one of us (E.S.) and cultured in the laboratory on low-salt agar plates (Lahl et al. 2003). *Panagrolaimus* sp. PS1159 is an unnamed parthenogenetic species, isolated in North Carolina by J. Millar. *Panagrolaimus* sp. ES5 is obligately outcrossing, and was isolated from dried blackberry twigs by E. Schierenberg in Bornheim, Germany. *Panagrolaimus superbus* DF5050 is also a male-female species, and was isolated from a gull's nest on the island of Surtsey, Iceland by B. Sohlenius. *Propanagrolaimus* sp. JU765, a protandrous hermaphrodite, was isolated by M.-A. Felix in Guangxi, China from a rice paddy. *Panagrolaimus* sp. DL137 was isolated by

D. Denver in Corvallis, Oregon from soil on University Campus, and *Panagrolaimus* sp. PS1579 was isolated by M.-A. Felix in Huntington Gardens, California. Cultures were usually kept at 15°C, but to quickly increase worm populations before DNA extraction, the culture temperature was increased to 25°C. To minimise confounding factors due to population divergence (or even the mixing of samples on one plate) single worms were picked, propagated for a few generations and then sequenced. Stabilates are stored anhydrobiotically at the Universität zu Köln. The parthenogenetic *P.* sp. PS1159 and the hermaphroditic *Prop.* sp. JU765 were bottlenecked for >30 generations in several single offspring lines, while the other 3 species (*P.* sp. ES5, *P. superbus*, *P. sp.* DAW1) were held in large populations and propagated by the transfer of several worms at a time. *Panagrolaimus sp.* DAW1 (previously designated *Panagrolaimus* sp. CB1 and *P. davidi* [Wharton et al. 2017]), was collected from the McMurdo Sound region, Antarctica by D. Wharton. Other tylenchine nematode genomes analysed were obtained from WormBase (www.wormbase.org).

Genome sizes and chromosome numbers

We estimated genome sizes in several isolates of *Panagrolaimus* using Feulgen image analysis densitometry (FIAD, following the protocols in (Hardie et al. 2016)), integrated optical densities (IODs) from at least 14 nuclei per individual worm were compared with erythrocytes of *G. domesticus* (C-value =1.25 pg) under x100 magnification (immersion oil, nD = 1.5150). The DNA content in picograms was converted to megabases using the formula 1 Mb = 1.022 * 10^-3 pg or 1 pg = 978 Mb (Dolezel et al. 2003). These measurements were complemented by RT-D-PCR assay (Wilhelm 2003) based on orthologues of *C. elegans* locus ZK682.5 (WBGene00022789), which has been identified as a nematode-wide single copy gene (Mitreva et al. 2011). Chromosomes were counted, after DAPI staining, in oocytes and 1-cell embryos. For this, gravid nematodes were picked from plates and transferred to M9 buffer and dissected with an insect needle to release gonadal tubes and embryos. These were transferred with a capillary tube into a small drop of 2μg/ml DAPI (Sigma) on a microscope slide, squashed under a coverslip in the stain and the preparation was sealed with nail varnish. After 10-15 min staining the preparations were analysed under an Olympus FluoView1000 confocal microscope with a 60x (NA 1.35) oil objective. Karyotypes were taken as valid only when confirmed in 10 independent chromosome sets. See supplementary fig. 1.

Genome Assembly

We constructed genome assemblies from Illumina short read libraries. After running first pass assemblies with the CLC Assembly Cell v.4.2 we applied the khmer pipeline (Brown et al. 2012) to digitally normalise read coverage on ES5, *P. superbus*, JU765 and PS1159 data. We identified and removed contaminating bacterial sequences using the blobtools approach (Kumar et al. 2013). The cleaned reads were reassembled using the Velvet assembler (Zerbino and Birney 2008) exploring different k-mer sizes. We then employed RNA-Seq derived mRNA predictions (see below) to scaffold the genomes with the SCUBAT pipeline (https://github.com/elswob/SCUBAT). For *P. superbus* the genome input to SCUBAT originated from a CLC assembly, as this proved better than the tested Velvet assemblies. For *P. sp.* DAW1, where the original assembly (Thorne et al. 2014) was of low completeness (39% complete KOGs as assessed by CEGMA), we (re-)assembled the genomes with SPAdes (Bankevich et al. 2012) and the redundans pipeline (Pryszcz and Gabaldón 2016). We also used SPAdes and the redundans pipeline to assemble the *P.* sp. PS1579 genome. Assembly qualities were evaluated with custom scripts. We utilized GNU Parallel (Tange 2011) in various steps of the assembly pipeline and in downstream analyses.

To assemble mitochondrial genomes contigs generated in the main genome assemblies were extended with Illumina reads using IMAGE (Tsai et al. 2010), then aligned to reference with ABACAS (Assefa et al. 2009) and, finally, any remaining gaps in the sequence filled with GapFiller (Boetzer and Pirovano 2012). All assembled mitochondrial genomes were annotated using the MITOS2 (Bernt et al. 2013) online pipeline. Additionally, the boundaries of protein coding genes (PCGs) in the new genomes were manually curated using the ORF finder tool (https://www.ncbi.nlm.nih.gov/orffinder/) and BLAST+ searches, while the tRNA predictions made by MITOS2 were verified using the online version of ARWEN (Laslett and Canbäck 2008). See supplementary fig 4 for a comparison of the mitochondrial genomes.

Transcriptome assembly

To aid gene annotation and to confirm expression of important genes we sequenced transcriptomes of mixed life cycle stages in *P.* sp. PS1159, *P.* sp. PS1579, *P.* sp. ES5, and *Prop.* sp. JU765 using Illumina TruSeq RNA-Seq. Raw reads were adaptor and quality trimmed with Trimmomatic (versions ≤0.32) before assembly with Trinity (Haas et al. 2013). We further sequenced the *de novo* transcriptomes of *P.* sp. DL137 (see also (Schiffer et al. 2014)). For *P. superbus* a transcriptome enriched for nematodes in the anhydrobiotic stage was generated using the Roche 454 platform and SMART cDNA synthesis. We assessed

the completeness of our transcriptome assemblies using CEGMA and also with BUSCO (through gVolante [Nishimura et al. 2017)) using the eukaryotic gene set as reference.

Repetitive DNA

We used a two-pronged approach to screen for repetitive elements in the final draft genomes. First we used RepeatModeler (open-1.0; http://www.repeatmasker.org/RepeatModeler.html) to identify and RepeatMasker (v. open-4.0.5) (Smit 2015) to mask genomes using the BLAST-based search engine. In addition we used *LTRharvest* (Ellinghaus et al. 2008) implemented in the GenomeTools analysis system (Gremme et al. 2013) to screen for transposons missed by RepeatModeler, but did not detect any additional transposons.

As genomes assembled from short insert libraries may collapse repeats into a single contig, we screened the genomes with a second, k-mer based assay to identify regions with high base coverage in the raw data that could be repetitive. Following a protocol established by Dan Bolser (unpublished; https://gitub.com/dbolser/PGSC/blob/master/kmer-filter/README), which relies on 'tallymer' from the *GenomeTools* package, we counted the frequency of all unique and repeated k-mers of sizes 10 - 50 bases. For each genome we then picked a k-mer size close to the value where frequency curves plateaued and counted the occurrence of k-mers at coverage levels 10 - 50. We identified the sequences in the genome corresponding to these k-mers and analysed the base coverage for these genomic regions based on CLC mappings with the aid of BEDtools (Quinlan and Hall 2010). We compared these empirical coverage distributions with genome wide coverage and selected an additional set of potential repeats to add to the RepeatModeler set. This unified set was then fed back into RepeatMasker for a second round of masking.

Gene Prediction

We used Augustus (v.3.0.1) (Stanke and Waack 2003) for gene prediction, employing an iterative approach to generate the most credible predictions. Augustus was trained with CEGMA-predicted genes. Wherever RNA-Seq data were available (*P.* sp. ES5, *P.* sp. PS1159, *Prop.* sp. JU765), it was assembled based on alignment to the genome, using gsnap (Wu and Nacu 2010), which has been shown to perform well in finding correct splice sites (Engström et al. 2013). These mappings then served as evidence for Augustus in a second round of gene prediction. For the first round of Augustus training in *P. superbus* we used exonerate (Slater and Birney 2005) to align predicted *P. sp.* PS1159 proteins, as no large-scale Illumina RNA-Seq data were available. We then mapped findorf (Krasileva et al.

2013) predicted ORFs (see below) from the 454-sequencing derived *P. superbus* transcriptome using BLAT (Kent 2002) and created Augustus evidence from this. For *P. sp.* DAW1 no transcriptomic data were available at the time of our analyses, and we used the *P.* sp. PS1159 species model in Augustus along with the *P. sp.* DAW1 CEGMA genes as hints. We implemented a best hit BLAST approach using different taxonomic databases to identify likely contamination in predicted genes. Candidate contamination was removed from the predicted gene sets and from the corresponding contigs in the genome assemblies. In the ES5 genome we identified an excess of *E. coli* contamination and employed mugsy (Angiuoli et al. 2011) for full genome alignments between the nematode and *E. coli* to identify and remove the corresponding contigs. Contamination was also additionally screened for in the HGT assay.

Open reading frames were predicted from *de novo* transcriptome assemblies of *P. superbus* (454-sequencing derived data, as well as DL137 and PS1579 (both high coverage Illumina RNA-Seq) using findorf. The findorf approach involved comparison of transcripts with proteomes from other nematode species (*Brugia malayi*, *Caenorhabditis briggsae*, *Caenorhabditis elegans*, *P.* sp. ES5, *Prop.* sp. JU765, *Meloidogyne hapla*, *Panagrellus redivius*, *P.* sp. PS1159, and preliminary data from *Plectus sambesii*) using BLASTX to identify frameshifts and premature stop codons, and hidden Markov models (HMMs) to identify Pfam domains to infer ORF start positions. In total, findorf inferred 21,381 *P. superbus* ORFs, 34,868 *P.* sp. DL137 ORFs and 47,754 *P.* sp. PS1579 ORFs.

Codon usage for the predicted genes was analysed with GCUA (McInerney 1998) and codonW (v.1.4.4; http://codonw.sourceforge.net). We also used codonW to compare codon usage between genes putatively gained through HGT and the genomic background of the host nematodes. We used tRNAscan-SE (v1.3.1) (Lowe and Eddy 1997) to identify tRNA genes, selecting the implemented Cove probabilistic RNA prediction package as the search engine.

Proteome Annotation

Putative domains in 13 nematode proteomes were inferred using InterProScan (v5.7-48.0) (Jones et al. 2014). We analysed all our *Panagrolaimus* species (including both transcriptomic and genomic data for *P. superbus*), our *Propanagrolaimus* outgroup, and the two remote outgroup species *Caenorhabditis elegans* and *Ascaris suum*. A summary of the Pfam domains annotation is deposited on the accompanying genome hubs webpage for the *Panagrolaimus* genomes.

Orthologous Proteins

We inferred orthology between proteins from our newly sequenced panagrolaimids and a set of outgroup species from the Tylenchina (Clade IV of Nematoda *sensu* Blaxter (Blaxter et al. 1998) using OrthoMCL (v.2.0.8) (Li et al. 2003), OrthoInspector (v.2.11) (Linard et al. 2015), and OrthoFinder (Emms and Kelly 2015). We also included data from the cephalobid species *Acrobeloides nanus* (Schiffer et al. 2018), as well as the model *C. elegans* (Rhabditina; Clade V) and *Ascaris suum* (Spirurina; Clade III). To detect orthologs to key *C. elegans* developmental genes (see section on GRNs) we primarily relied on the extensively tested and robust OrthoMCL pipeline based on NCBI blast, which we validated with OrthoInspector. The latter found additional divergent orthologous, which were hidden to OrthoMCL. OrthoFinder based on Diamond (Buchfink et al. 2014) BLAST was only used in the GRAMPA (Thomas et al. 2016) analysis, since the output contains gene trees for each cluster (see section on ploidy below). In cases were OrthoMCL and OrthoInspector grossly disagreed or the presence/absence pattern across species appeared inconsistent in the GRN analysis, we directly screened genomes for the presence of a gene using the HMM profile figmop pipeline (Curran et al. 2014).

While our use of additional orthology finding programs and the figmop pipeline added confidence to the inference of absence of genes, it is still possible that orthologous genes were not detected due to rapid sequence evolution in Nematoda. Thus, we wanted to implement an additional test: It had previously been shown that using a synteny approach some particularly fast evolving genes in the sex determination pathway of *Caenorhabditis* species can be identified, as collinearity is maintained (Kuwabara and Shah 1994, Streit et al. 1999, Haag et al. 2002). Consequently, we performed a synteny analysis with MCScanX (Wang et al. 2012). For this we compared the genomes of *Panagrolaimus* PS1159, *Propanagrolaimus* JU765, which had the best contiguity of our newly assembled genomes and included the *Bursaphelenchus xylophilus* genome as an independent control. We used lenient (non-standard) parameters (evalue:1e-03 (default: 1e-5); match size: 2 (d: 5); maximum distance: 10 (d: 5)) in MCScanX, and as a proof of principle also compared the genomes of *C. elegans*, *C. briggsae*, and *C. remanei*.

Phylogenetic analyses

To increase confidence in the phylogenetic positions of species in our analyses we extended an existing phylogeny (Lewis et al. 2009) by adding all 452 proteins predicted by the CEGMA pipeline. We constructed individual alignments for each cluster of orthologous CEGMA proteins from our species and outgroups with Clustal Omega (v1.2) (Sievers et al.

2011) and then used trimAl (v1.4.rev15) (Capella-Gutiérrez et al. 2009) to exclude ambiguous regions. A supermatrix combining all trimmed alignments was then constructed with the aid of phyutility (v2.2.6) (Smith and Dunn 2008).

Phylogenies were inferred using the MPI version of PhyloBayes (1v.4f) (Lartillot et al. 2013) with the CAT model implemented therein and RAxML (v7.7.2) (Stamatakis 2006) using the VT model under the GAMMA parameter, as predicted by Prottest (v3.2) (Darriba et al. 2011). We let RAxML automatically stop the bootstrap replicates sampling needed to reach convergence and it found 56 trees after 861 bootstraps. Bayesian analyses in the MPI version of PhyloBayes did not converge on a single tree after more than 8,000 generations in four parallel chains. This was due to *P. sp.* DAW1 swapping between two positions within the group of parthenogenetic *Panagrolaimus* species. However, tree topologies of both methods are otherwise congruent. Phylogenetic inferences for single proteins, or groups of orthologous (e.g. sHSP), were conducted with Clustal Omega, trimAl, protest3, and RAxML as described for the CEGMA KOGs. We employed Jalview (Waterhouse et al. 2009) and SeaView (Galtier et al. 1996) to visualise alignments, and used SplitsTree4 (Huson and Bryant 2005) to explore relationships in gene families. The phylogenetic inference was carried out using the CHEOPS cluster at the University of Köln.

Assessment of ploidy

We first mapped reads against the assembled small subunit ribosomal sequences in each species and identified variants. This suggested the presence of a significant "minor allelic variant" in the parthenogenetic species. Both the hermaphroditic species *Prop.* sp. JU765, expected to be diploid, and the potentially polyploid parthenogenetic strain *P.* sp. PS1159, were inbred through 30 generations of single offspring propagation; from this we expected genomes to be largely homogenised. In contrast, *P. sp.* DAW1 was expected to be more heterozygous as large populations from multiple plates were used for DNA extraction. Including the parthenogens *Acrobeloides nanus* (cephalobida, clade IV) and *Plectus sambesii* (plectica, clade III), where genomes (but not of sexual sister species) became recently available (Rosic et al. 2018; Schiffer et al. 2018), into this analysis, we aimed to gain a more general insight into the origin of parthenogenesis in *Nematoda.* We extracted all Augustus predicted genes for each species and mapped RNA-Seq read sets against the coding sequences using the CLC mapper (v.5.0), requiring a sequence identity of 90% and a read length threshold of 80%. We performed the same mapping approach with genomic reads against repeat masked and unmasked genomes. We used BamTools (Barnett et al. 2011) (v.2.3.0) to sort the mappings, SAMtools (v1.0-13) (Li et al. 2009) to create mpileups,

VarScan (v2.3.6) (Koboldt et al. 2012) to call variants, and collated the minor variant frequency spectrum (folded site spectrum) for each species. We then implemented a two sample Kolmogorov Smirnov (KS) test available in the Julia (Bezanson et al. 2012) programming language to analyse whether the observed variant frequencies are different under the null hypotheses that the frequencies are sampled from the same distribution (Supplementary Table 1): we first sampled 20,000 data points from each distribution and directly compared these with the KS test; next we ran 50,000 bootstrap replicates on 20,000 randomly mixed samples per distribution, and then measured the exact p-value in the bootstrapped distribution.

For the GRAMPA analysis testing allo- vs. auto-polyploidy we used all ~15k FastME trees from running Orthofinder mid-point rooted with NOTUNG (v2.8.1.7) (Chen et al. 2000). To validate these results we also used a more comprehensive initial dataset including the DL137 and PS1579 proteomes and selected 2313 groups of orthologues from OrthoMCL that had a 2:1 ratio of parthenogenetic to sexual species. For each group we built an alignment with clustal-omega, inferred a protein tree with RAxML and ran GRAMPA. This resulted in the same topology as the OrthoFinder FastME based GRAMPA tree shown in Fig 1.

Estimation of divergence times

We used ANDI to calculate pairwise evolutionary distances between three species of *Panagrolaimus* (*P. superbus, P.* sp. ES5, and *P.* sp. PS1159) and two outgroups (*Propanagrolaimus* sp. JU765, and *Panagrellus redivivus*) (see supplementary table 2). We excluded the parthenogenetic *P. sp.* DAW1 from this analysis since the assembly span of 118 Mb and our variant calling (see above) indicate this assembly to contain duplicate regions when compared to the quasi-haploid assembly of PS1159 (85Mb). ANDI is designed for comparisons of entire genomes, and does not require homologous sequences or genes as input. To convert ANDI-distances into divergence measured in generations or in absolute time we used the divergence of *Caenorhabditis briggsae* and *Caenorhabditis* sp. 5 as a reference. The *Caenorhabditis* species are separated by about 171 M generations (i.e. 85.5 M generations since their last common ancestor (Cutter 2008)). Generation time measurements were conducted by Isabel Goebel at the University of Köln (unpublished thesis "Untersuchungen zum Lebenszyklus von Nematoden mit unterschiedlichem Fortpflanzungsmodus") under supervision of PHS and ES. Briefly, nematodes from parthenogenetic strains *P.* sp. PS1159, and *P.* sp. PS1579, and the sexual species *P. superbus*, *P.* sp. ES5, and *Prop.* sp. JU765 were cultured at room temperature on small *E.*

coli-seeded agar plates. These were monitored every few hours during early development and then every day during adult life. Across all species assayed, generation times (Vancoppenolle et al. 1999) averaged 8 days under our laboratory conditions. Since there is some uncertainty in the generation length in nature we estimated timings based on 4, 8, 16, 32, and 50 days per generation (corresponding to around 91, 46, 231, 111 and 7 generations per year, respectively).

Identification of conserved protein domains with significantly different abundances in Panagrolaimid and other nematodes

We identified Pfam domain annotations in the proteomes of our species set using InterProScan. Statistical differences in abundance of individual Pfam domains was analysed using a two-sided Fisher's exact test (Null Hypothesis Significance Testing - NHST), corrected for multiple testing using the Benjamin-Hochberg algorithm (for false discovery rate) implemented in the R (R Core Team) statistical language. We also analysed transcriptome assemblies from *P.* sp. DL137 (for which we have no genome) and *P.* sp. PS1159 (to retrieve proteins potentially missed in the genome-wide annotations).

To support and further investigate the results of the NHST with an independent method, we employed classifier analysis in a support vector machine (SVM) framework to address two pivotal questions:

(1) Do the patterns of annotation frequency derived from InterProScan correspond to defining differences between species groups?

(2) Are the annotations that together form separate and clearly identifiable patterns for each group associated with functions that differ between them?

Given a number of samples, each belonging to one of *x* possible classes, a classifier attempts to learn the underlying sample-class mapping. Classes are labels with (in the present case) three possible values (*Panagrolaimus*, *Propanagrolaimus, Panagrellus*). Samples were n-dimensional vectors of annotation presence and absence. The classifier was trained on a set of samples, including an equal number of representatives from both classes and then tested on its ability to predict the class of an independent sample. We used 30 randomly drawn annotations from each representative species as dimensions in the classifier and ran 2000 iterations, each time selecting a different subset of training samples and test species. We tested variations relying on 50 or 100 dimensions (annotations), but found that these did not change the overall pattern of results. The analyses based on thirty dimensions are presented.

Classification of Panagrolaimidae achieved 55 % accuracy (standard error of the mean [SEM] = 0.006; chance level classification = 50%) by an SVM based on randomly selecting 30 Pfam domains in each of 2000 iterations. The same approach yielded 58% mean accuracy (SEM = 0.006) classification for the Outgroup; the difference between classification performance for Panagrolaimidae and Outgroup was not statistically significant in a two-samples t-test ($T(1999) < 1$), showing that the classifier was not biased towards one class. More importantly, classification performance was significantly better than control classification (where sample-class mapping in the training set was permuted). Based on a permuted class-sample mapping in the training set, the classifier achieved a mean accuracy for Panagrolaimidae of 49 % (SEM = 0.006) and of 49% for the Outgroup (SEM = 0.008). The comparison between the real classifier incorporating true sample-to-class mappings in the training set and the permuted classifier was statistically significant for Panagrolaimidae: $T = 7$, $p < 0.001$, $df = 1999$; and for the outgroup: $T= 7.4$, $p < 0.001$, $df = 1999$ (see supplementary fig. 5). During classification, we recorded annotations that were most often part of successful classification iterations. These lists were then intersected with results from NHST analysis and interpreted with reference to identifying differences in the biology of each species group.

Detection of Horizontal Gene Transfers

To detect candidate horizontal gene transfers (HGT), we used Alienness (Rancurel et al. 2017) to calculate an Alien Index (AI) (Gladyshev et al. 2008; Flot et al. 2013). Briefly, all Panagrolaimidae predicted proteins were compared against the NCBI's nr protein database using BLAST (Altschul et al. 1990) with an E-value threshold of $1e^{-3}$ and no SEG filtering. BLAST hits were parsed to retrieve associated taxonomic information, using the NCBI taxonomy database. For every Panagrolaimidae protein returning at least one hit in both metazoan and non-metazoan species, we calculated the AI:

$$AI = \ln(\text{best metazoan E-value} + E^{-200}) - \ln(\text{best non-metazoan E-value} + E^{-200})$$

When either no significant metazoan or non-metazoan BLAST hit was found, a penalty E-value of 1 was automatically assigned. To detect HGT events that took place in an ancestor of Panagrolaimidae or its close relatives, BLAST hits to Panagrolaimoidea (TaxID: 55746) and Aphelenchina (TaxID: 1182516) were excluded from the calculation of the AI. An AI > 0 indicates a better hit to a non-metazoan species than to a metazoan species and thus a possible acquisition *via* HGT. An AI > 30 corresponds to a difference of magnitude $1.0e^{-10}$ between the best non-metazoan and best metazoan E-values and is taken as strong indication of a HGT event. All *Panagrolaimidae* proteins that returned an AI > 0 and that

aligned with ≥70 % identity to a non-metazoan protein were considered as possible contaminants and were discarded from the analysis. Potential candidates were further validated by identifying gene models that contained spliceosomal introns, were surrounded by *bona fide* nematode genes, had mapped RNA-Seq reads (TPM estimates were calculated with RSEM [Li and Dewey 2011] incorporating bowtie2 [Langmead and Salzberg 2012] mapping) and had codon usages similar to bulk genome values (supplementary fig. 6).

Reconstruction of the timing of gene acquisitions *via* HGT

We used Mesquite (v3.01) (Maddison and Maddison 2018) to reconstruct the timing of gene acquisition *via* HGT. By adding orthology information to HGT candidates, we built a matrix of presence/absence of each HGT candidate across the different *Panagrolaimidae* species. We mapped this matrix to the species phylogeny, including two additional outgroups (*Acrobeloides nanus* and *Bursaphelenchus xylophilus*). Based on presence/absence information, Mesquite was used to trace back ancestral presence / absence at each node, using a parsimony model. When ancestral presence/absence of a gene family at a given node was equally parsimonious, we arbitrarily considered the family as present, because secondary loss of a gene acquired by HGT ancestrally is intuitively more likely than proposing multiple independent HGT events. HGT candidates that were species-specific were considered as acquired specifically in this species.

Functional analysis of HGT candidates

Pfam annotations were retrieved for all HGT candidate proteins, and domains that were conserved among the set of candidate HGT proteins of several Panagrolaimidae species identified (Supplementary Excel file HGT – PFAM). We also predicted functions based on the presence of Pfam domains, using pfam2go (available at geneontology.org/external2go/pfam2go) and custom perl scripts. To make Gene Ontology annotations comparable, we mapped raw GO terms to the generic GOslim ontology. We used the GOSlimViewer, developed as part of AgBase (McCarthy et al. 2006), to map terms to the GOslim ontology (Supplementary Excel file HGT – GOSlim).

Mitochondrial genome assembly and annotation

To assemble mitochondrial genomes contigs generated in the main genome assemblies were extended with Illumina reads using IMAGE (Tsai et al. 2010), then aligned to reference

with ABACAS (Assefa et al. 2009) and, finally, any remaining gaps in the sequence filled with GapFiller (Boetzer and Pirovano 2012).

All assembled mitochondrial genomes were annotated using the MITOS2 (Bernt et al. 2013) online pipeline. Additionally, the boundaries of protein coding genes (PCGs) in the new genomes were manually curated using the ORF finder tool (https://www.ncbi.nlm.nih.gov/orffinder/) and BLAST+ searches, while the tRNA predictions made by MITOS2 were verified using the online version of ARWEN (Laslett and Canbäck 2008). See supplementary Fig 4 for a comparison of the mitochondrial genomes.

Codon usage

Codon usage for the predicted genes was analysed with GCUA (McInerney 1998) and codonW (v.1.4.4; http://codonw.sourceforge.net). We also used codonW to compare codon usage between genes putatively gained through HGT (see below) and the genomic background of the host nematodes. We used tRNAscan-SE v1.3.1 (Lowe and Eddy 1997) to identify tRNA genes, selecting the implemented Cove probabilistic RNA prediction package as the search engine.

Signatures of adaptive evolution

We used Crann (Creevey and McInerney 2003) to explore signatures of selection in panagrolaim genes. Over 20,000 OrthoMCL derived orthology cluster protein alignments, built using clustal-omega, were reverse-translated into DNA alignments with trimAl and SAMtools. We retained 7,335 alignments that contained at least one parthenogenetic and one male female *Panagrolaimus* species. Further filtering of short and outlier sequences within each of these gene family alignments, using amino acid versions of the sequence alignments, removed poorly-defined clusters. If the length of any sequence was less than half the length of the overall alignment it was removed. For all remaining sequences in each alignment, two filtering steps were carried out: A distance matrix was constructed from the amino acid versions of the alignments using ProtDist from the PHYLIP (Felsenstein 1993) package and the JTT model of evolution was used to construct a neighbour-joining tree with neighbour from the PHYLIP package. Using custom scripts, the average taxon-taxon path length on the tree was calculated, and outlier sequences which had a significantly greater average path length were identified using R. These sequences were removed from both the DNA and amino acid alignments. The second filter checked for saturation in the nucleotide versions of the alignment. This was carried out by building two trees with PAUP for each alignment, the first using a P distance and the second using the HKY model. The taxon-

taxon length across both trees were calculated as before and both calculations combined for visualization as a scatter plot, using R. Any alignments exhibiting signatures of saturation following visual inspection of the plots were removed from both the DNA and amino acid alignments. The resulting filtered DNA alignments were input to Crann to calculate all pairwise rates of non-synonymous substitutions per non-synonymous site (dN) and synonymous substitution per synonymous site (dS). The average of all pairwaise dN/dS ratios between the following categories of species were calculated: (1) sexually-reproducing *Panagrolaimus* species versus parthogenetic *Panagrolaimus* species; (2) all *Panagrolaimus* species, versus nearest outgroups; (3) all species in analysis versus all. See supplementary fig. 10. The rates of dN/dS were sorted into categories by their functions (Tatusov et al. 1997) (supplementary fig. 9).

Supplementary References

Altschul SF, Fish W, Miller W, Myers EW, Lipman DJ. 1990. Basic local alignment search tool. J Molec. Biol. 3:403-410.

Angiuoli SV, Dunning H JC, Salzberg SL, Tettelin H. 2011. Improving pan-genome annotation using whole genome multiple alignment. BMC Bioinformatics 12:272.

Assefa S, Keane TM, Otto TD, Newbold C, Berriman M. 2009. ABACAS: algorithm-based automatic contiguation of assembled sequences. Bioinformatics 25:1968–1969.

Bankevich A, Nurk S, Antipov D, et al. 2012. SPAdes: a new genome assembly algorithm and its applications to single-cell sequencing. J Comput Biol. 19:455–477.

Barnett DW, Garrison EK, Quinlan AR, Strömberg MP, Marth GT. 2011. BamTools: a C++ API and toolkit for analyzing and managing BAM files. Bioinformatics 27:1691–1692.

Bernt M, Donath A, Jühling F, Externbrink F, Florentz C, Fritzsch G, Pütz J, Middendorf M, Stadler PF 2013. MITOS: improved de novo metazoan mitochondrial genome annotation. Mol Phyl. Evol 69:313–319.

Bezanson J, Karpinski S, Shah VB, Edelman A. 2012. Julia: A Fast Dynamic Language for Technical Computing. arXiv.

Blaxter ML, De Ley P, Garey JR, Le Guédard M, Hanano A, Heintz D, Ehlting J, Herrfurth C, Feussner I, Bessoule J-J. 1998. A molecular evolutionary framework for the phylum Nematoda. Nature 392:71–75.

Boetzer M, Pirovano W. 2012. Toward almost closed genomes with GapFiller. Genome Biol 13:R56.

Brown CT, Howe A, Zhang Q, Pyrkosz AB, Brom TH. 2012. A single pass approach to reducing sampling variation, removing errors, and scaling de novo assembly of shotgun sequences. arXiv.

Buchfink B, Xie C, Huson DH. 2014. Fast and sensitive protein alignment using DIAMOND. Nat Methods. 12:59–60.

Capella-Gutiérrez S, Silla-Martínez JM, Gabaldón T. 2009. TrimAl: a tool for automated alignment trimming in large-scale phylogenetic analyses. Bioinformatics 25:1972–1973.

Chen K, Durand D, Farach-Colton M. 2000. NOTUNG: a program for dating gene duplications and optimizing gene family trees. J Comput Biol. 7:429–447.

Curran DM, Gilleard JS, Wasmuth JD. 2014. Figmop: a profile HMM to identify genes and bypass troublesome gene models in draft genomes. Bioinformatics 30:3266–3267.

Cutter AD. 2008. Divergence times in Caenorhabditis and Drosophila inferred from direct estimates of the neutral mutation rate. Mol Biol Evol 25:778–786.

Darriba D, Taboada GL, Doallo R, Posada D. 2011. ProtTest 3: fast selection of best-fit models of protein evolution. Bioinformatics 27:1164–1165.

Davies PL. 2014. Ice-binding proteins: a remarkable diversity of structures for stopping and starting ice growth. Trends in Biochemical Sciences 39:548–555.

Eki T, Ishihara T, Katsura I, Hanaoka F. 2007. A genome-wide survey and systematic RNAi-based characterization of helicase-like genes in Caenorhabditis elegans. DNA Res. 14:183–199.

Ellinghaus D, Kurtz S, Willhoeft U. 2008. LTRharvest , an efficient and flexible software for de novo detection of LTR retrotransposons. BMC Bioinformatics 9:18.

Emms DM, Kelly S. 2015. OrthoFinder: solving fundamental biases in whole genome comparisons dramatically improves orthogroup inference accuracy. Genome Biol. 16:E9–E13.

Engström PG, Steijger T, Sipos B, Grant GR, Kahles A, The RGASP Consortium, Rätsch G, Goldman N, Hubbard TJ, Harrow J, Guigó R, Bertone P. 2013. Systematic evaluation of spliced alignment programs for RNA-seq data. Nat Methods. 10:1185–1191.

Felsenstein J. 1993. Phylogenetic Inference Programs (PHYLIP).

Flot J-F, Hespeels B, Li X, Noel B, Arkhipova I, Danchin EGJ, Hejnol A, Henrissat B, Koszul R, Aury J-M, Barbe V, Barthelemy R-M, Bast J, Bazykin GA, Chabrol O, Couloux A, Da Rocha M, Da Silva C, Gladyshev E, Gouret P, Hallatschek O, Hecox-Lea B, Labadie K, Lejeune B, Piskurek O, Poulain J, Rodriguez F, Ryan JF, Vakhrusheva OA, Wajnberg E, Wirth B, Yushenova I, Kellis M, Kondrashov AS, Mark Welch DB, Pontarotti P, Weissenbach J, Wincker P, Jaillon O, Van Doninck K. 2013. Genomic evidence for ameiotic evolution in the bdelloid rotifer Adineta vaga. Nature 500:1–5.

Galtier N, Gouy M, Gautier C. 1996. SEAVIEW and PHYLO_WIN: two graphic tools for sequence alignment and molecular phylogeny. Comput Appl Biosci 12:543–548.

Garofalo A, Rowlinson M-C, Amambua NA, et al. 2003. The FAR protein family of the nematode *Caenorhabditis elegans*. Journal of Biological Chemistry 278:8065–8074.
Gladyshev EA, Meselson M, Arkhipova IR. 2008. Massive horizontal gene transfer in bdelloid rotifers. Science 320:1210–1213.
Gremme G, Steinbiss S, Kurtz S. 2013. GenomeTools: A comprehensive software library for efficient processing of structured genome annotations. IEEE/ACM Transactions on Computational Biology and Bioinformatics (TCBB) 10:645–656.
Haag ES, Wang S, Kimble J. 2002. Rapid coevolution of the nematode sex-determining genes fem-3 and tra-2. Curr Biol 12:2035–2041.
Haas BJ, Papanicolaou A, Yassour M, Grabherr M, Blood PD, Bowden J, Couger MB, Eccles D, Li B, Lieber M, MacManes MD, Ott M, Orvis J, Pochet N, Strozzi F, Weeks N, Westerman R, William T, Dewey CN,Henschel R, LeDuc RD, Friedman N, Regev A. 2013. De novo transcript sequence reconstruction from RNA-Seq: reference generation and analysis with Trinity. Nat Protoc. 8:1494–1512.
Hara, R, C P Selby, M Liu, D H Price, and A Sancar. 1999. Human transcription release factor 2 dissociates RNA polymerases I and II stalled at a cyclobutane thymine dimer. Journal of Biological Chemistry 274: 24779–24786. doi:10.1074/jbc.274.35.24779.
Hardie DC, Gregory TR, Hebert PDN. 2016. From pixels to picograms: A beginners' guide to genome quantification by Feulgen image analysis densitometry. Journal of Histochemistry & Cytochemistry 50:735–749.
Heger, Peter, Michael Kroiher, Nsah Ndifon, and Einhard Schierenberg. 2010. Conservation of MAP kinase activity and MSP genes in parthenogenetic nematodes. BMC Dev Biol: 51. doi: 10.1186/1471-213X-10-51.
Hespeels B, Knapen M, Hanot-Mambres D, Heuskin AC, Pineux F, Lucas S, Koszul R, van Doninck K. 2014. Gateway to genetic exchange? DNA double-strand breaks in the bdelloid rotifer *Adineta vaga* submitted to desiccation. J of Evol Biol 27:1334–1345.
Huson DH, Bryant D. 2005. Estimating Phylogenetic Trees and Networks Using SplitsTree 4. www.splitstree.org
Jones P, Binns D, Chang H-Y, Fraser M, Li W, McAnulla C, McWilliam H, Maslen J, Mitchell A, Nuka G, Pesseat S, Quinn AF, Sangrador-Vegas A, Scheremetjew M, Yong S-Y, Lopez R, Hunter S. 2014. InterProScan 5: genome-scale protein function classification. Bioinformatics 30:1236–1240.
Karim N, Jones JT, Okada H, Kikuchi T. 2009. Analysis of expressed sequence tags and identification of genes encoding cell-wall-degrading enzymes from the fungivorous nematode *Aphelenchus avenae*. BMC Genomics 2009 10:1 10:525.
Kent WJ. 2002. BLAT---The BLAST-Like Alignment Tool. Genome Res 12:656–664.
Koboldt DC, Zhang Q, Larson DE, Shen D, McLellan MD, Lin L, Miller CA, Mardis ER, Ding L, Wilson RK. 2012. VarScan 2: Somatic mutation and copy number alteration discovery in cancer by exome sequencing. Genome Res 22: 568–576.
Krasileva KV, Buffalo V, Bailey P, Pearce S, Ayling S, Tabbita F, Soria M, Wang S, Consortium IWGS,
Akhunov E, Uauy C, Dubcovsky J. 2013. Separating homeologs by phasing in the tetraploid wheat transcriptome. Genome Biol 14:R66.
Kumar S, Jones M, Koutsovoulos G, Clarke M, Blaxter M. 2013. Blobology: exploring raw genome data for contaminants, symbionts and parasites using taxon-annotated GC-coverage plots. Front. Genet. 4.
Kuwabara PE, Shah S. 1994. Cloning by synteny: identifying C. briggsae homologues of C. elegans genes. Nucleic Acids Res 22:4414–4418.
Lahl V, Halama C, Schierenberg E. 2003. Comparative and experimental embryogenesis of Plectidae (Nematoda). Dev Genes Evol. 213:18–27.
Langmead B, Salzberg SL. 2012. Fast gapped-read alignment with Bowtie 2. Nat Methods. 9:357–359.
Lartillot N, Rodrigue N, Stubbs D, Richer J. 2013. PhyloBayes MPI: phylogenetic reconstruction with infinite mixtures of profiles in a parallel environment. Systematic Biol 62:611–615.
Laslett D, Canbäck B. 2008. ARWEN: a program to detect tRNA genes in metazoan mitochondrial nucleotide sequences. Bioinformatis 24:172–175.
Lewis SC, Dyal LA, Hilburn CF, Weitz S, Liau W-S, Lamunyon CW, Denver DR. 2009. Molecular evolution in *Panagrolaimus* nematodes: origins of parthenogenesis, hermaphroditism and the Antarctic species *P. davidi*. BMC Evol Biol 9:15.

Li B, Dewey CN. 2011. RSEM: accurate transcript quantification from RNA-Seq data with or without a reference genome. BMC Bioinformatics 12:323.
Li H, Handsaker B, Wysoker A, et al. 2009. The Sequence Alignment/Map format and SAMtools. Bioinformatics 25:2078–2079.
Li L, Stoeckert CJ, Roos DS. 2003. OrthoMCL: identification of ortholog groups for eukaryotic genomes. Genome Res 13:2178–2189.
Lowe TM, Eddy SR. 1997. tRNAscan-SE: a program for improved detection of transfer RNA genes in genomic sequence. Nucleic Acids Res 25:955–964.
Lynch M, Bürger R, Butcher D, Gabriel W. 1993. The mutational meltdown in asexual populations. J Heredity 84:339–344.
Linard B, Allot A, Schneider R, Morel C, Ripp R, Bigler M, Thompson JD, Poch O, Lecompte O. 2015. OrthoInspector 2.0: Software and database updates. Bioinformatics 31:447–448.
Maddison, W. P. and D.R. Maddison. 2018. Mesquite: a modular system for evolutionary analysis. Version 3.51. http://www.mesquiteproject.org.
McCarthy FM, Wang N, Magee GB, Nanduri B, Lawrence ML, Camon EB, Barrell DG, Hill DP, Dolan ME, Williams WP, Luthe DS, Bridges SM, Burgess SC. 2006. AgBase: a functional genomics resource for agriculture. BMC Genomics 2013 14:1 7:229.
McInerney JO. 1998. GCUA: general codon usage analysis. Bioinformatics 14:372–373.
Mitreva M, Jasmer DP, Zarlenga DS, Wang Z, Abubucker S, Martin J,Taylor CM, Yin Y, Fulton L, Minx P, Yang S-P, Warren WC, Fulton RS, Bhonagiri V, Zhang X, Hallsworth-Pepin K, Clifton SW, McCarter JP,Appleton J, Mardis ER, Wilson RK. 2011. The draft genome of the parasitic nematode *Trichinella spiralis*. Nat Genetics. 43:228–235.
Muller HJ. 1964. The relation of recombination to mutational advance. Mut. Research 1:2–9.
Nishimura O, Hara Y, Kuraku S. 2017. gVolante for Standardizing Completeness Assessment of Genome and Transcriptome Assemblies. Bioinformatics 22: 3635–3637.
Pryszcz LP, Gabaldón T. 2016. Redundans: an assembly pipeline for highly heterozygous genomes. Nucleic Acids Res 44:e113–e113.
Quinlan AR, Hall IM. BEDTools: a flexible suite of utilities for comparing genomic features. Bioinformatics 26: 841–842.
Rancurel C, Legrand L, Danchin E. 2017. Alienness: rapid detection of candidate horizontal gene transfers across the tree of life. Genes 8:248.
Rosic S, Amouroux R, Requena CE, Gomes A, Emperle M, Beltran T, Rane JKm Linnett S, Selkirk ME, Schiffer PH, Bancroft AJ, Grencis RK, Jeltsch A, Hajkova P, Sarkies P. 2018. Evolutionary analysis indicates that DNA alkylation damage is a byproduct of cytosine DNA methyltransferase activity. Nat Genetics. 50:1–12.
Schiffer PH, Nsah NA, Grotehusmann H, Kroiher M, Loer C, Schierenberg E. 2014. Developmental variations among Panagrolaimid nematodes indicate developmental system drift within a small taxonomic unit. Dev. Genes Evol. 224:183–188.
Schiffer PH, Gravemeyer J, Rauscher M, Wiehe T. 2016. Ultra large gene families: a matter of adaptation or genomic parasites? Life 6.
Schiffer PH, Polsky AL, Cole AG, Camps JIR, Kroiher M, Silver DH, Grishkevich V, Anavy L, Koutsovoulos G, Hashimshony T, Yanai I. 2018. The gene regulatory program of *Acrobeloides nanus* reveals conservation of phylum-specific expression. Proc Natl Acad Sci 1:201720817–201724464.
Schmitz C, Kinge P, Hutter H. 2007. Axon guidance genes identified in a large-scale RNAi screen using the RNAi-hypersensitive Caenorhabditis elegans strain Nre-1(Hd20) Lin-15b(Hd126). Proc Natl Acad Sci 104: 834–839.
Schwarz EM, Kato M, Sternberg PW. 2012. Functional transcriptomics of a migrating cell in *Caenorhabditis elegans*. Proc Natl Acad Sci 109: 16246–16251. doi:10.1073/pnas 1203045109.
Sievers F, Wilm A, Dineen D, Gibson TJ, Karplus K, Li W, Lopez R, McWilliam H, Remmert M, Soeding J, Thompson JD, Higgins DG. 2011. Fast, scalable generation of high-quality protein multiple sequence alignments using Clustal Omega. Mol. Syst. Biol. 7:539–539.
Smit AFA. RepeatMasker Open-4.0. 2015. www.repeatmasker.org.
Smith SA, Dunn CW. 2008. Phyutility: a phyloinformatics tool for trees, alignments and molecular data. Bioinformatics 5:715–716.

Srinivasan J, Dillman AR, Macchietto MG, et al. 2013. The draft genome and transcriptome of *Panagrellus redivivus* are shaped by the harsh demands of a free-living lifestyle. Genetics 193:1279–1295.
Stamatakis A. 2006. RAxML-VI-HPC: maximum likelihood-based phylogenetic analyses with thousands of taxa and mixed models. Bioinformatics 22:2688–2690.
Stanke M, Waack S. 2003. Gene prediction with a hidden Markov model and a new intron submodel. Bioinformatics 19:215–ii225.
Stricker SA. 1999. Comparative biology of calcium signaling during fertilization and egg activation in animals. Dev Biol 211:157–176.
Streit A, Li W, Robertson B, Schein J, Kamal IH, Marra M, Wood WB. 1999. Homologs of the *Caenorhabditis elegans* masculinizing gene her-1 in *C. briggsae* and the filarial parasite *Brugia malayi*. Genetics 152:1573–1584.
Tange O. 2011. Gnu parallel-the command-line power tool. The USENIX Magazine
Tatusov RL, Koonin EV, Lipman DJ. 1997. A genomic perspective on protein families. Science 278:631–637.
Thomas GWC, Ather SH, Hahn MW. 2016. Gene-tree reconciliation with MUL-trees to resolve polyploidy events. Systematic Biol. 66:1007-1018.
Thorne MAS, Kagoshima H, Clark MS, Marshall CJ, Wharton DA. 2014. Molecular analysis of the cold tolerant Antarctic nematode, *Panagrolaimus davidi*. PLoS ONE 9:e104526.
Thorne MAS, Seybold A, Marshall C, Wharton D. 2017. Molecular snapshot of an intracellular freezing event in an Antarctic nematode. Cryobiology 75:117–124.
True JR, Haag ES. 2001. Developmental system drift and flexibility in evolutionary trajectories. Evolution & Development 3:109–119.
Tsai IJ, Otto TD, Berriman M. 2010. Improving draft assemblies by iterative mapping and assembly of short reads to eliminate gaps. Genome Biol 11:R41.
Vancoppenolle B, Borgonie G, Coomans A. 1999. Generation times of some free-living nematodes cultured at three temperatures. Nematology 1:15–18.
Wang Y, Tang H, Debarry JD, Tan X, Jingping L, Wang X, Tae-ho L, Jin H, Marler B, Guo H, Kissinger JC, Paterson AH. 2012. MCScanX: a toolkit for detection and evolutionary analysis of gene synteny and collinearity. Nucleic Acids Res 40:e49–e49.
Waterhouse AM, Procter JB, Martin DMA, Clamp M, Barton GJ. 2009. Jalview Version 2--a multiple sequence alignment editor and analysis workbench. Bioinformatics 25:1189–1191.
Wharton, DA, Marshall CJ, Egeter B. 2017. Comparisons between two Antarctic nematodes: cultured *Panagrolaimus* sp. DAW1 and field-sourced *Panagrolaimus davidi*. Nematology:19, 533–542.
Wu TD, Nacu S. 2010. Fast and SNP-tolerant detection of complex variants and splicing in short reads. Bioinformatics 26:873–881.
Xu XZS, Sternberg PW. 2003. A C. elegans sperm TRP protein required for sperm-egg interactions during fertilization. Cell 114:285–297.
Zerbino DR, Birney E. 2008. Velvet: algorithms for de novo short read assembly using de Bruijn graphs. Genome Res 18:821–829.

Supplementary Figure 1

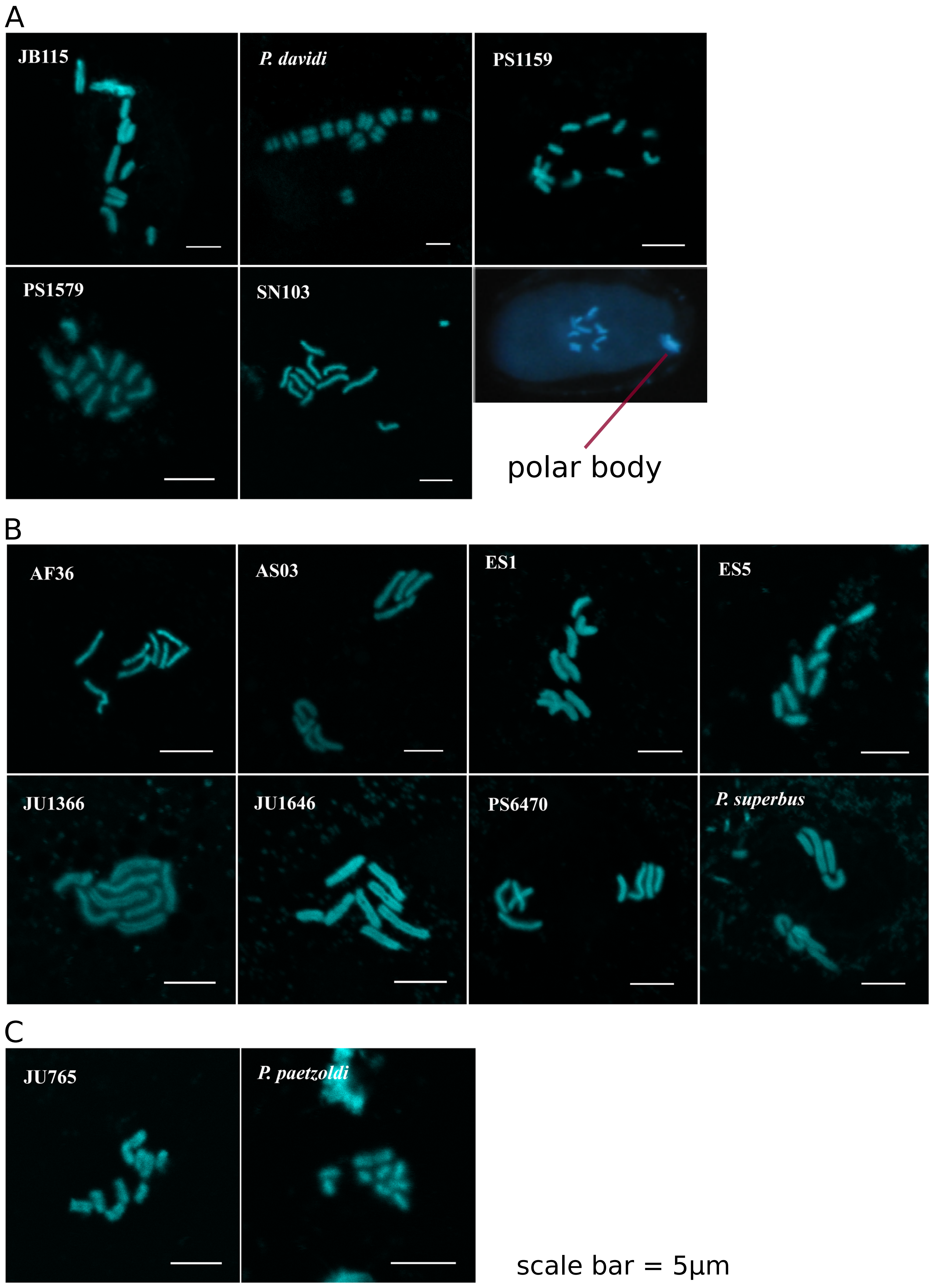

Supplementary Figure 2

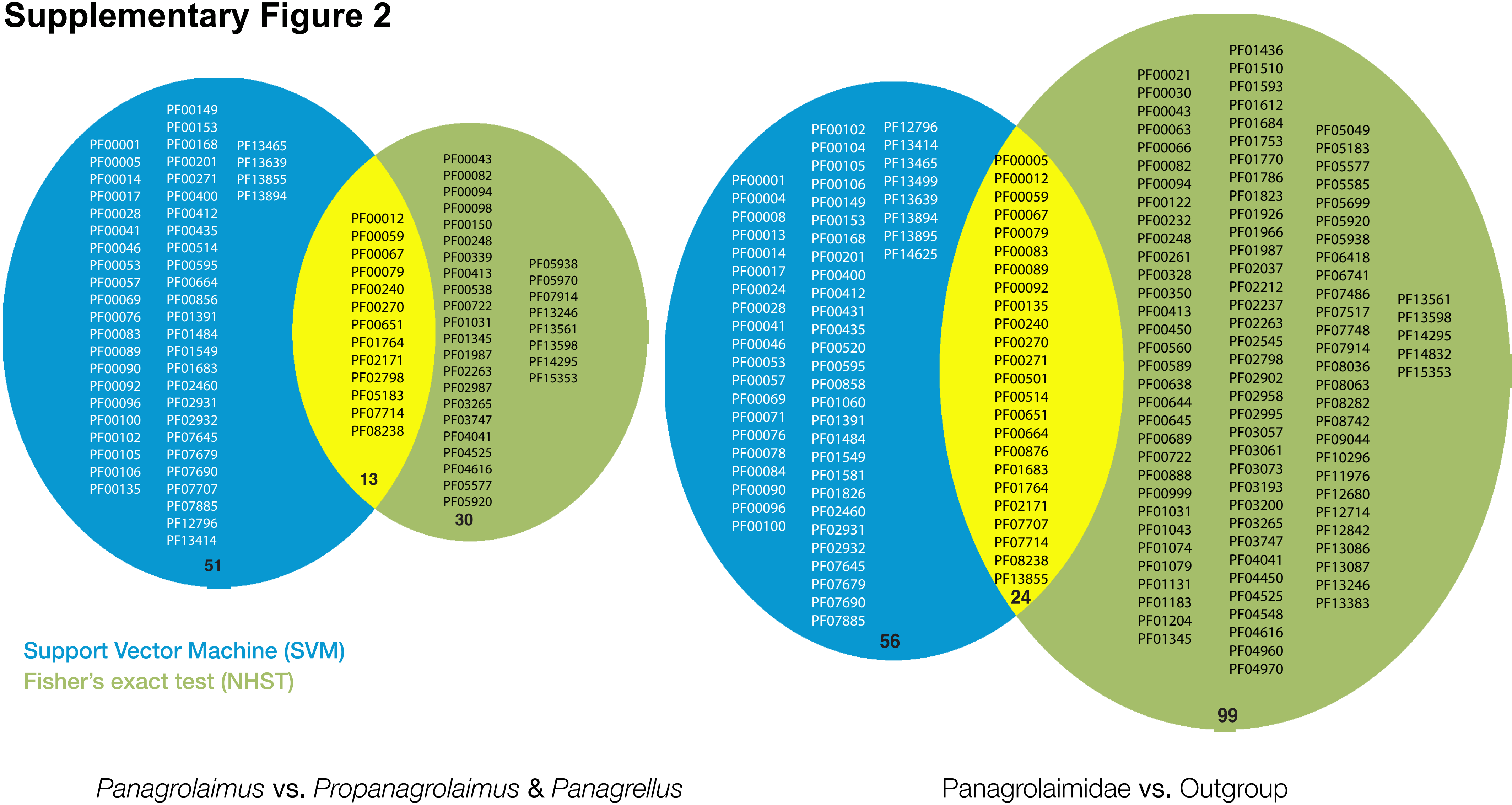

Supplementary Figure 3

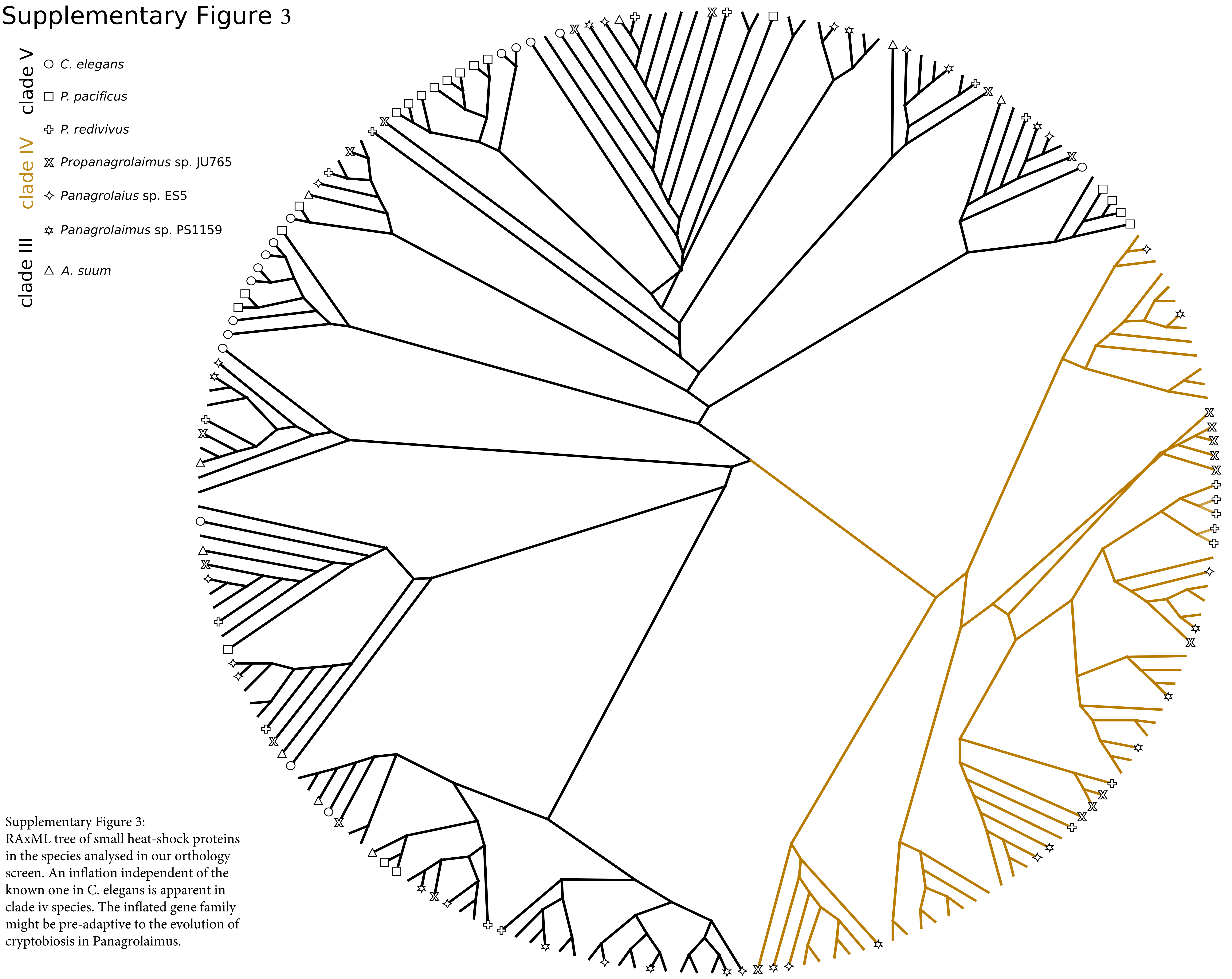

Supplementary Figure 3:
RAxML tree of small heat-shock proteins in the species analysed in our orthology screen. An inflation independent of the known one in C. elegans is apparent in clade iv species. The inflated gene family might be pre-adaptive to the evolution of cryptobiosis in Panagrolaimus.

Supplementary Figure 4

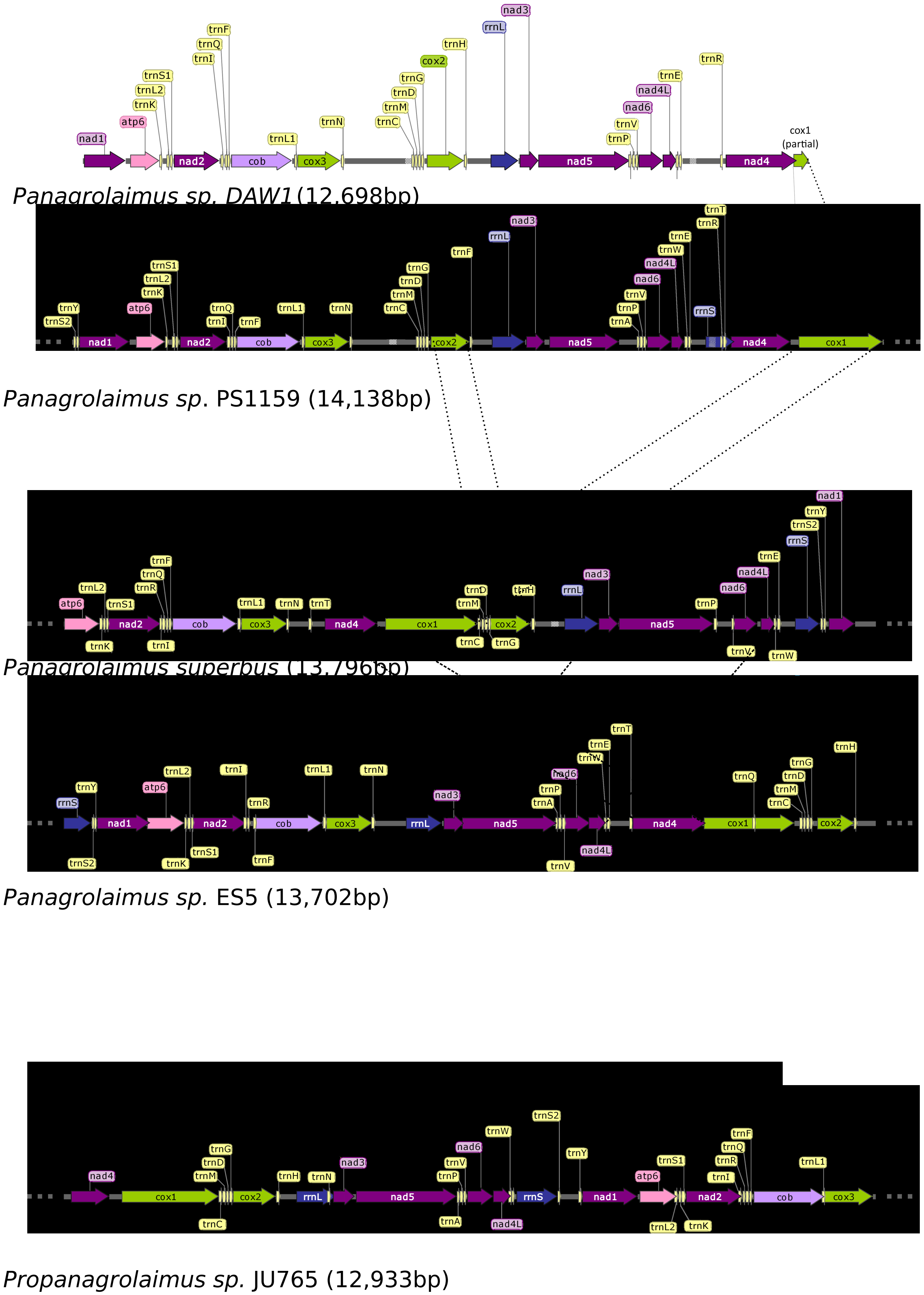

Supplementary Figure 5

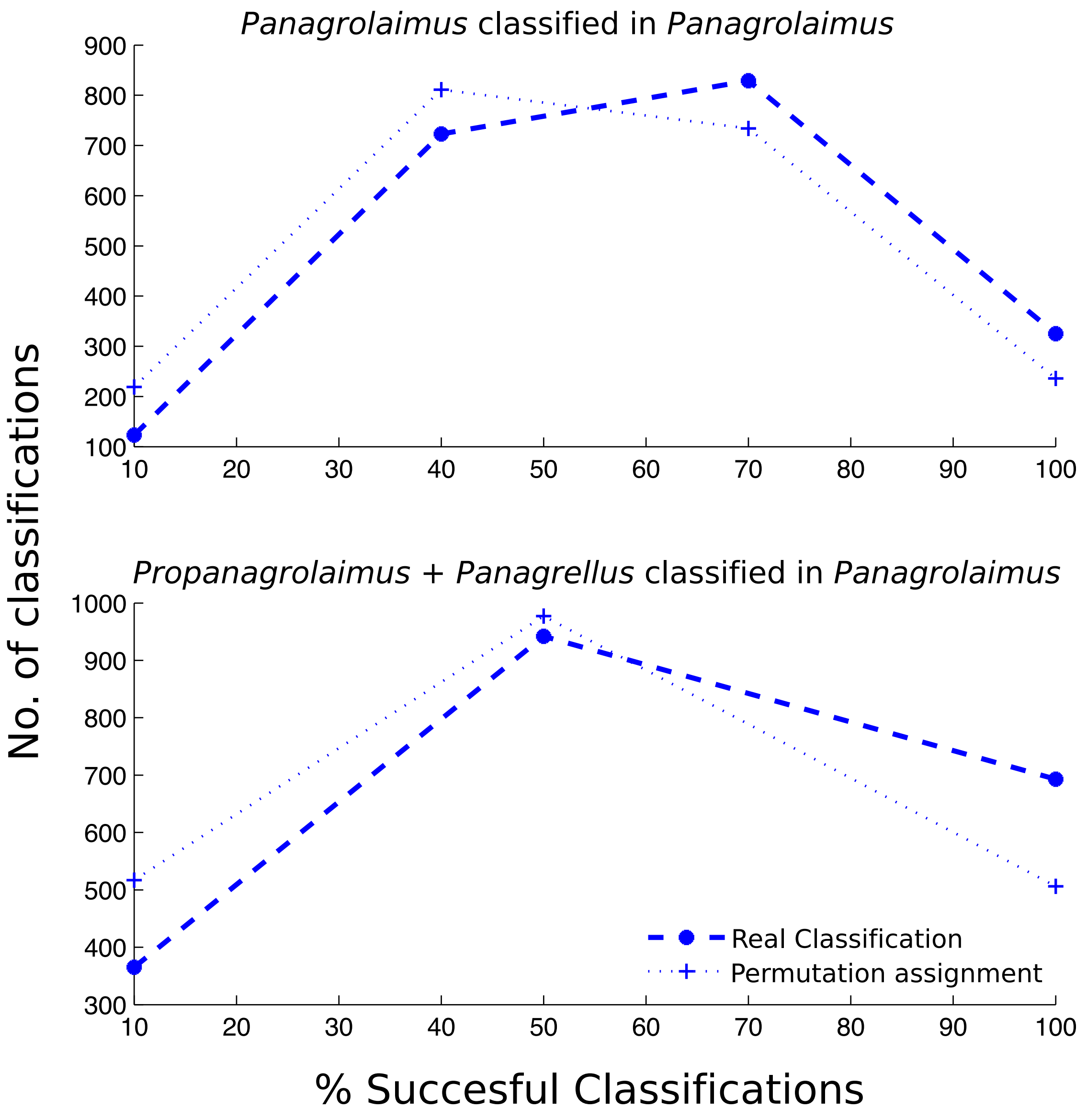

PS1159

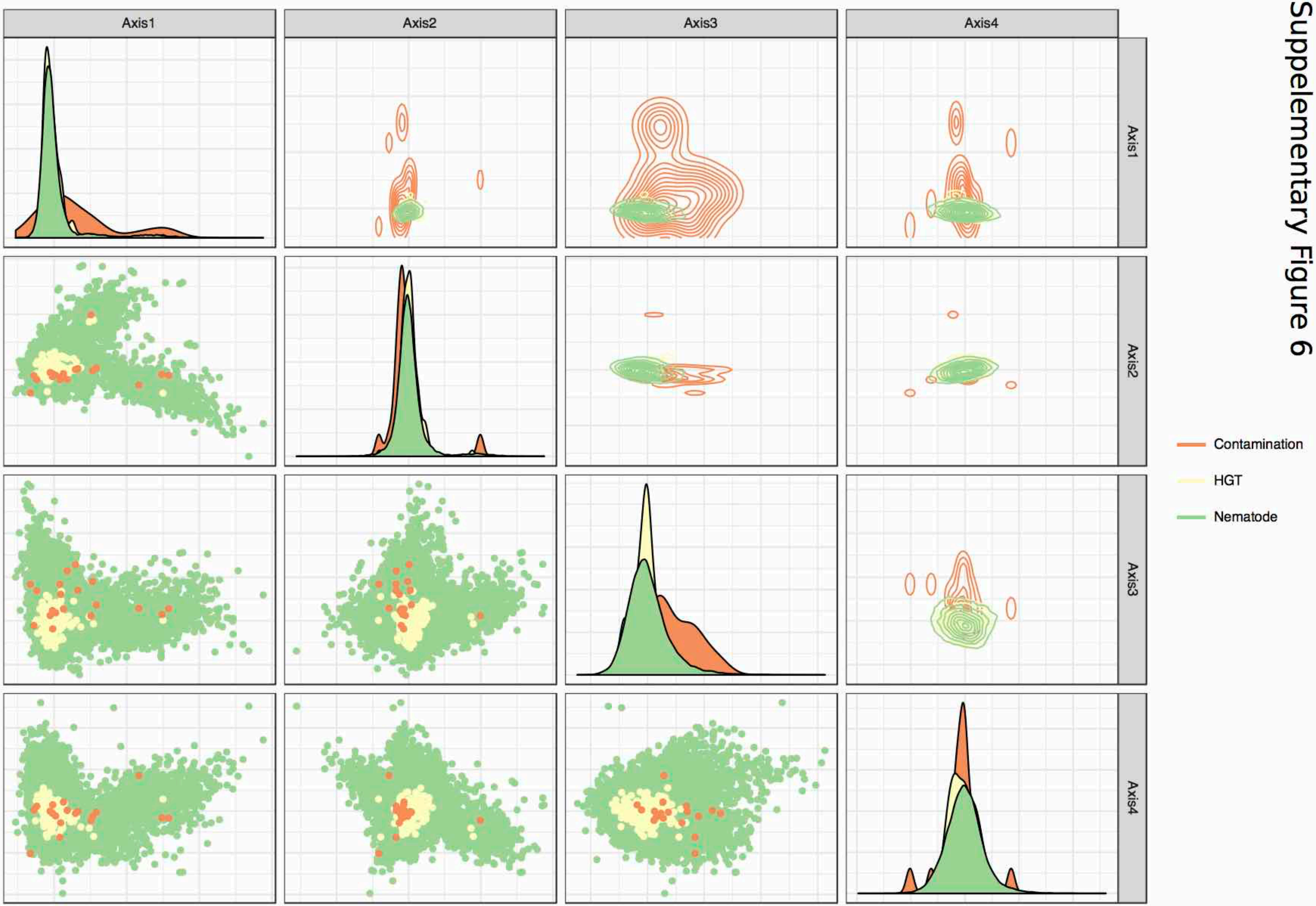

Suppelementary Figure 6

Supplementary Figure 7

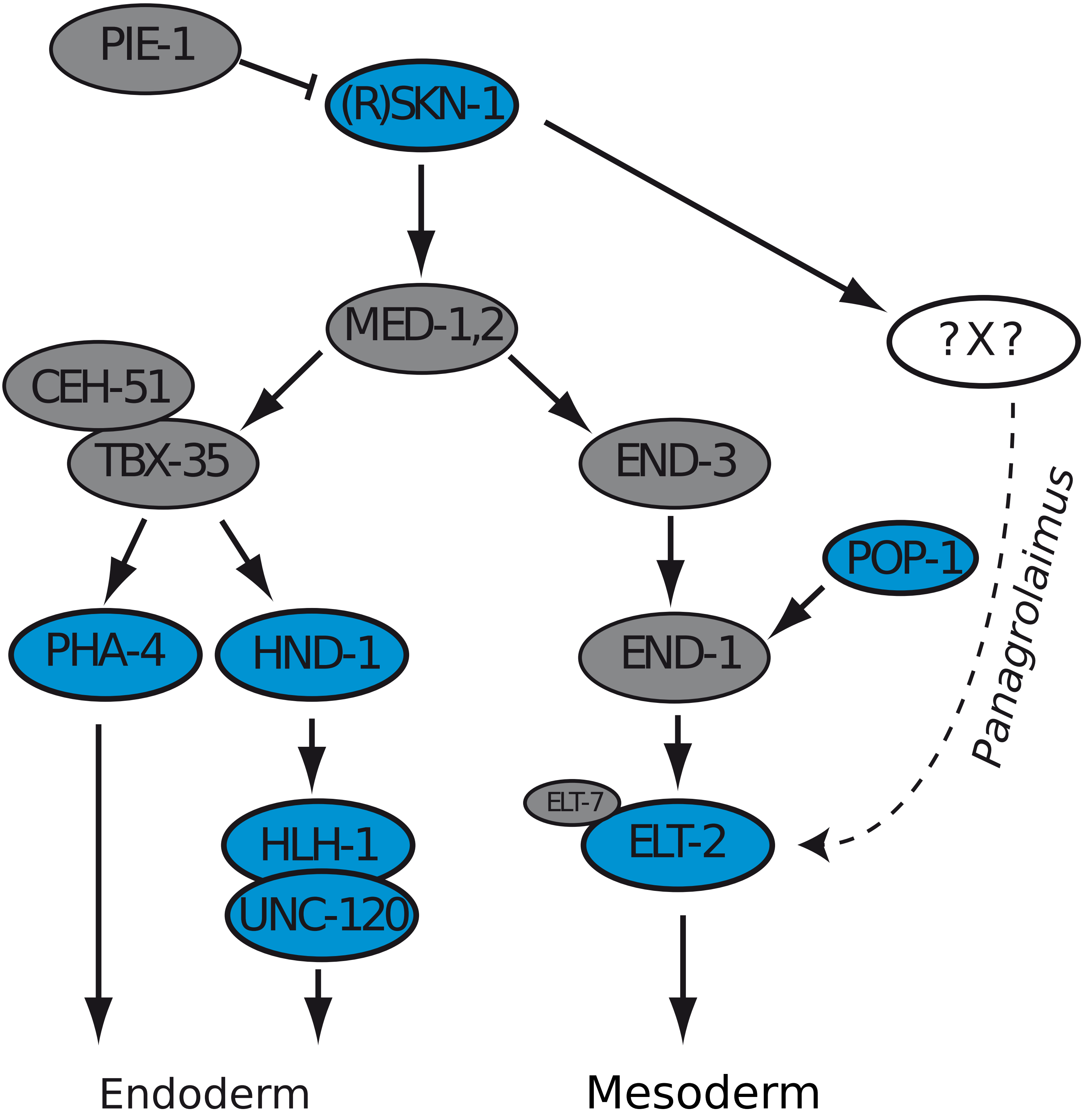

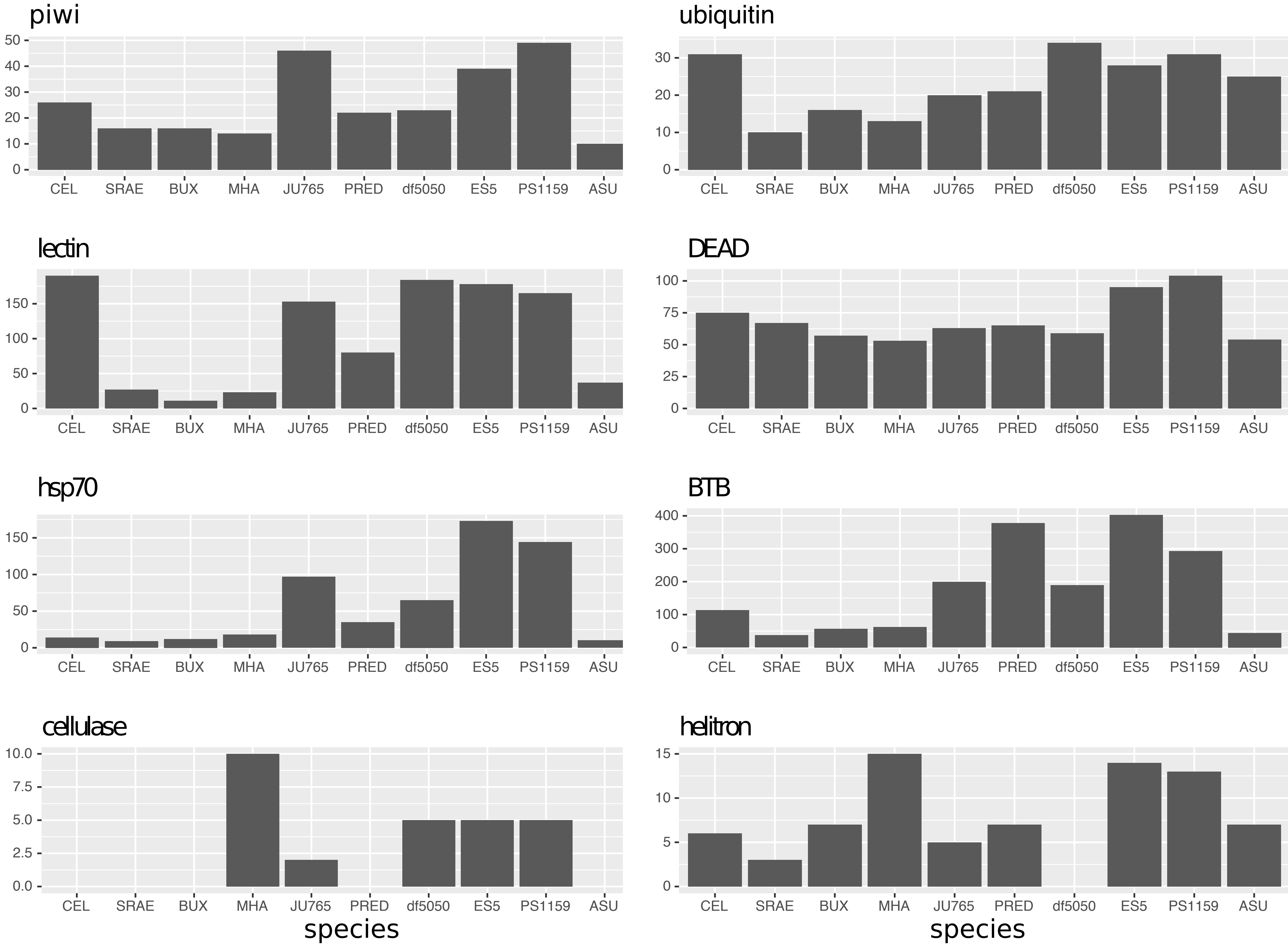

Supplementary Figure 8

Supplementary Figure 9

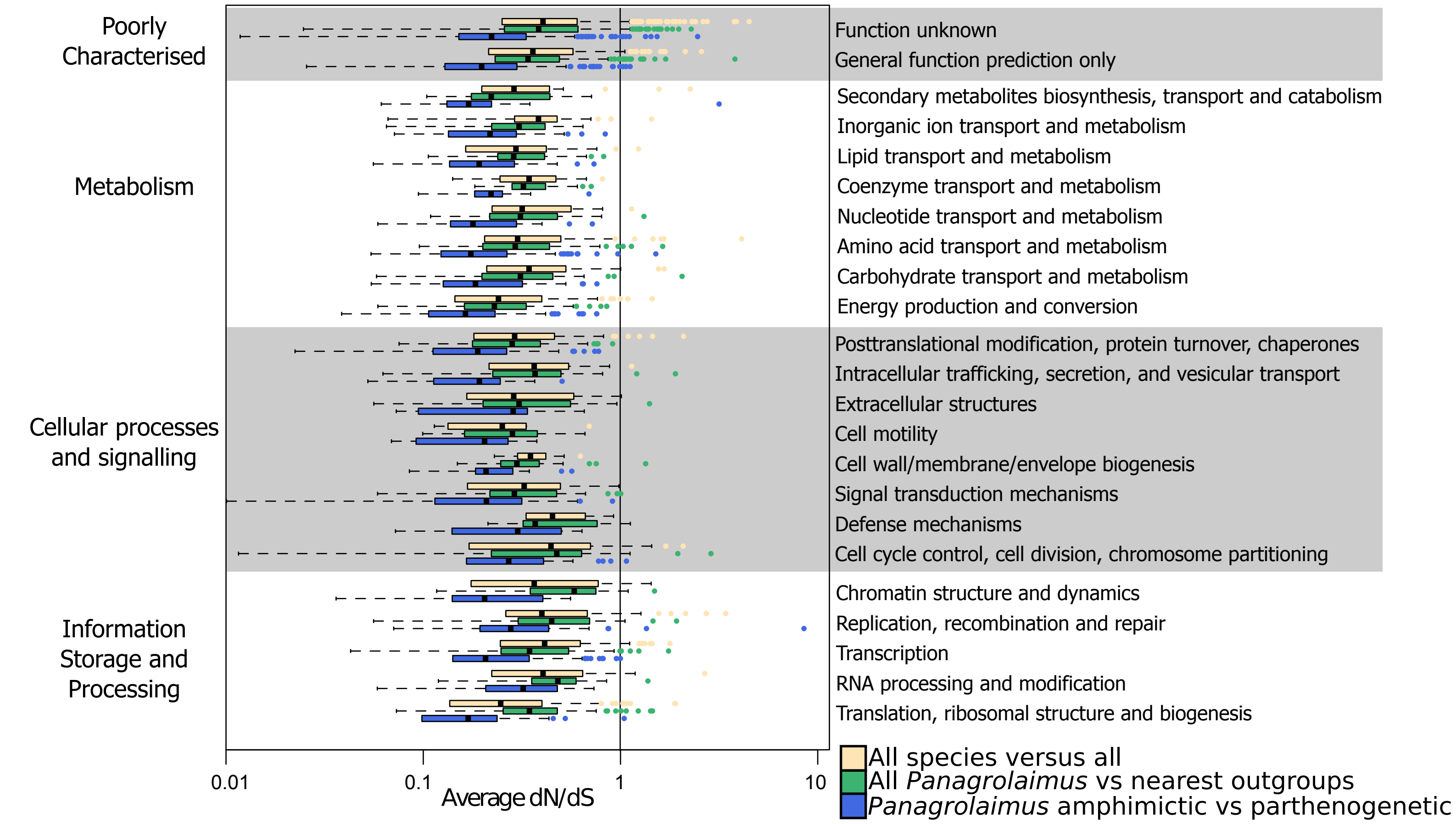

Supplementary Figure 10A

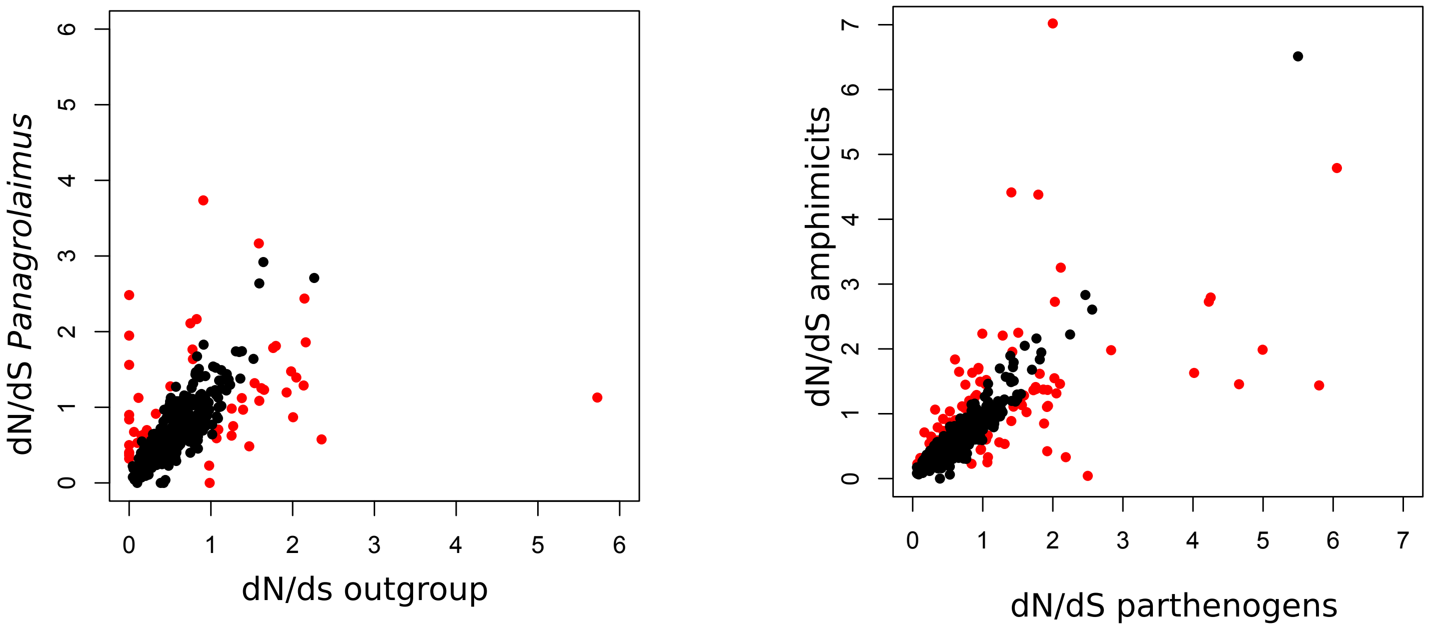

Supplementary Figure 10:

A. Plots of dN/dS values showing outlier genes (in red) for comparisons between cryptobiotic Panagrolaimus species and non-cryptobiotic outgroups and between parthenogenetic and amphictic Panagrolaimus species.

Supplementary Figure 10B

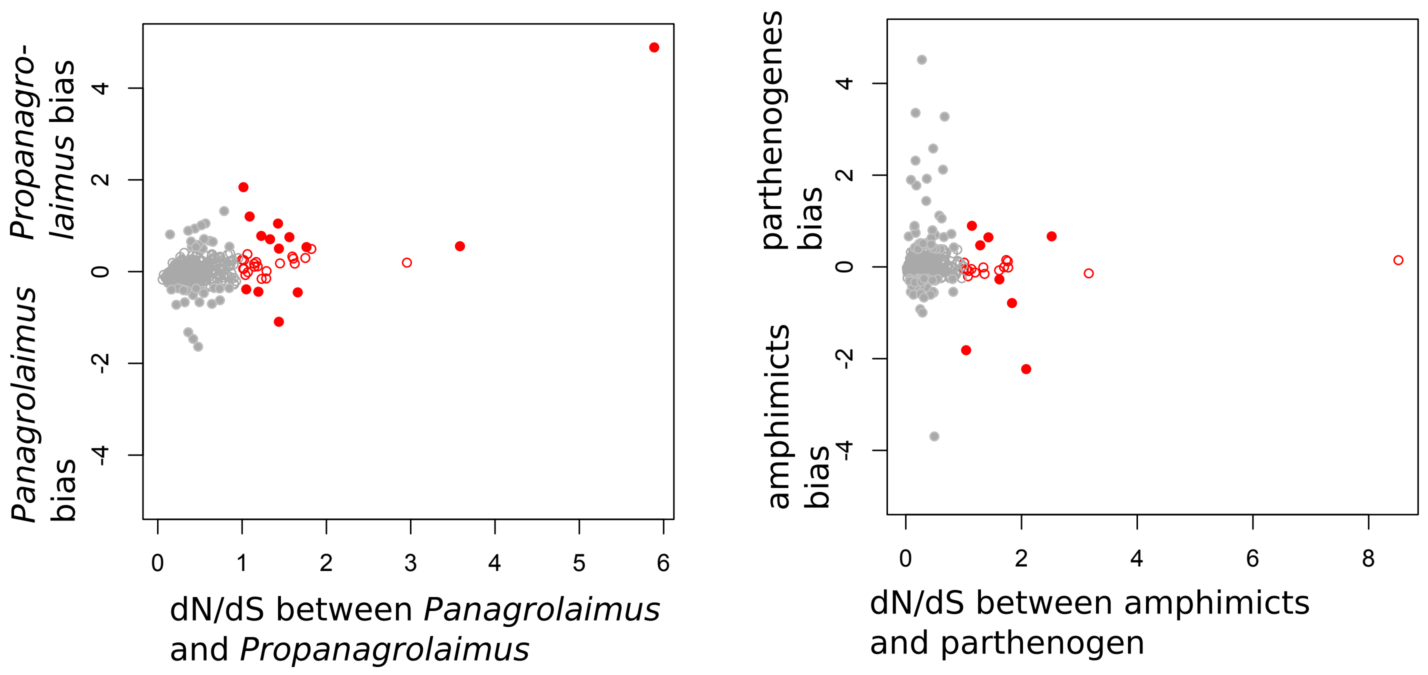

Supplementary Figure 10:

B. Plots displaying genes with bias towards a given taxon in both comparisons displayed in A.

Supplementary Figure Captions

Supplementary Figure 1:
Karyograms of parthenogenetic (**A**) and amphimictic (**B**) *Panagrolaimus* species, and outgroup species (**C**). All analysed amphimictic *Panagrolaimus* were found to have 2n=8, while the parthenogenetic had (xn=) 12 chromosomes. The *Propanagrolaimus* outgroup species had 2n=10 chromosomes, which is in line with the number in *Panagrellus redivivus*. Scale bar represents 5 μm. We observed the parthenogenetic *Panagrolaimus* species to have one polar body at the end of oocyte development, confirming meiosis.

Supplementary Figure 2:
Using a Fisher's exact test (green) and a Support Vector Machine classification (blue) we compared Pfam annotations. Firstly (left), to find genes potentially important in *Panagrolaimus* specific traits (e.g. desiccation tolerance) we tested species in the genus *Pangrolaimus* against (non-desiccating) *Propanagrolaimus* and *Panagrellus* (combined). Secondly (right), to search for panagrolaimidae traits we combined *Pangrolaimus* with *Propanagrolaimus* and *Panagrellus* and tested against outgroup species. Overall, we found several protein families associated with cryptobiosis, e.g HSPs, helicases, and C-type lectins, to be important for *Panagrolaimus* biology. See supplementary Excel table for Pfam description.

Supplementary Figure 3:
RAxML tree of small heat-shock proteins in the species analysed in our orthology screen. An inflation independent of the known one in *C. elegans* is apparent in clade IV species. The inflated gene family might be pre-adaptive to the evolution of cryptobiosis in *Panagrolaimus*.

Supplementary Figure 4:
Maps of the complete mitochondrial genomes of *Panagrolaimus superbus*, *Panagrolaimus* sp. ES5, *Panagrolaimus* sp. PS1159 and *Propanagrolaimus* sp. JU765. The *P. sp.* DAW1 mt genome could not be fully assembled, and PS1579 (not shown) remained in 4 fragments. The non-coding and unresolved portions of the genome are represented by continuous grey and shaded regions, respectively. All genes are encoded in the same direction (as indicated by arrow heads). Proteins and rRNA genes have standard nomenclature, tRNA genes are designated by single-letter abbreviations. Two tRNA genes are present for leucine (L1 and L2) and serine (S1 and S2). Note the different placement of the *cox* genes between PS1159 and *P. sp.* DAW1, and the other species, and the *nad5* gene between *P. superbus* and *P.* sp. ES5.

Supplementary Figure 5:
Histograms on the percent of correct classifications across all iterations of the classifier and classifier control permutation.

A. In this comparison of *Panagrolaimus* species each classification attempt can assign the correct label to either none, one, two, or three worms (samples). Comparisons show that the classifier trained on real sample-class mappings outperforms the control permutation significantly (trained on random assignment of samples to class labels), with a larger number of above-chance classifications (i.e., correct categorizations for 2, or 3 out of three worms).
B. In the comparison of outgroup species and Panagrolaims classification at chance level corresponds to correctly classifying one out of two worms. Accordingly, this is where the control permutation analysis peaks, whereas the classifier trained on correct class-to-sample mapping significantly outperforms the control, classifying both worms correctly in a larger number of iterations.

Supplementary Figure 6:
Plots showing the distribution of codon usage in *Panagrolaimus* sp. PS1159 genes identified as HGT candidates (orange) in comparison to genes identified as stemming from contamination (yellow) with aid of our AlienIndex analysis, and the genomic background (green). HGT candidates appear to be more similar in their codon usage to the genomic background, than the ones identified as contamination. Similar patterns have been observed in the other species and are indicative of an integration into the genomic background of the laterally acquired genes.

Supplementary Figure 7:
Many orthologues of *C. elegans* developmental GRNs could not be detected in either *Panagrolaimus* nor other clade IV (nor clade III) species in our analysis, indicating non-orthologues gene displacement and high evolutionary turn-over. One example displayed here, is the *C. elegans* endo-mesoderm specification GRN (missing genes in grey). It is possible that the induction of mesoderm formation was under direct influence of SKN-1, and particular WNT-pathway control, in species ancestral to clades IV and V. See Supplementary Results and Discussion for details.

Supplementary Figure 8:
Key gene families that were found to be inflated in *Panagrolaimus* species. Numbers are based on a screen of PFAM domains obtained by InterProScan followed by enrichment analysis (Fisher's test) comparing with *Propanagrolaimus* and *Panagrellus* as outgroups. Among others the displayed families have a potential role in the evolution of cryptobiosis in the *Panagrolaimus* species. Species abbreviations are: CEL – *C. elegans* (clade V), SRAE – *S. ratti*, BUX – *B. xylophylus*, MHA – *M. hapla*, JU765 – *Propanagrolaimus* sp. JU765, PRED – *P. redivivus*, DF5050 – *P. superbus*, ES5 – *P.* sp. ES5, PS1159 – *P.* sp. PS1159 (all preceding clade IV), ASU – *A. suum* (clade III).

Supplementary Figure 9:
Average rates of evolution (dN/dS) are shown for comparisons between parthenogenic and gonochoristic *Panagrolaimus* (in blue), *Panagrolaimus* and the closest outgroups

(*Propanagrolaimus* and *Panagrellus*) (in green), and between all species (in yellow) grouped by functional categories (see Supplementary Excel file dNdS). We find that while the majority of genes have a ratio of < 1 there are notable exceptions across several functional categories, both between parthenogenetic and gonochoristic species and between panagrolaimids and their closest relatives. Genes in these categories could play roles in the evolution of cryptobiosis and potentially also in the evolution of parthenogenesis.

Supplementary Figure 10:

A. Plots of dN/dS values showing outlier genes (in red) for comparisons between cryptobiotic *Panagrolaimus* species and non-cryptobiotic outgroups and between parthenogenetic and amphictic *Panagrolaimus* species.

B. Plots displaying genes with bias towards a given taxon in both comparisons displayed in A.

Supplementary Table 2: Pairwise divergence time estimates (in Myr) in *Panagrolaimus* spp. assuming a range of 4 to 50 days per generation. For calibrating divergence time we used the species pair *C. briggsae* and *C. sp5* a per base substitution rate of 0.1322 (calculated with Andi). Furthermore, we assumed a divergence time of 171M generations between these two species.
Note: divergence times from the common ancestor (Figure 2 B) are obtained by multiplying the numbers in this Table by the factor 0.5.

				4 days	8 days	16 days	32 days	50 days
Species A	repro	Species B	repro	Myr (4d/g = 91.2 gen/yr)	Myr (8 days/gen = 45.6 gen/yr)	Myr (16d/g = 22.8 gen/yr)	Myr (32d/g = 11.4 gen/yr)	Myr (50 days/gen = 7.3 gen/yr)
P. superbus	A	ES5	A	1.01	2.03	4.06	8.12	12.69
PS1159	P	ES5	A	1.32	2.65	5.29	10.58	16.53
PS1159	P	*P. superbus*	A	1.37	2.74	5.48	10.95	17.12
ES5	A	*P. redivivus*	A	1.34	2.68	5.35	10.71	16.73
PS1159	P	*P. redivivus*	A	1.49	2.98	5.95	11.91	18.61
P. superbus	A	*P. redivivus*	A	1.86	3.71	7.43	14.86	23.21
ES5	A	JU765	H	2.40	4.79	9.59	19.18	29.96
PS1159	P	JU765	H	2.42	4.83	9.66	19.32	30.19
P. superbus	A	JU765	H	2.52	5.05	10.10	20.20	31.56
P. redivivus	A	JU765	H	3.26	6.51	13.02	26.05	40.70
